# Proteomic features of gray matter layers and superficial white matter of the rhesus monkey neocortex: comparison of prefrontal area 46 and occipital area 17

**DOI:** 10.1007/s00429-024-02819-y

**Published:** 2024-06-28

**Authors:** Paola B. Castro-Mendoza, Christina M. Weaver, Wayne Chang, Maria Medalla, Kathleen S. Rockland, Lisa Lowery, Elizabeth McDonough, Merina Varghese, Patrick R. Hof, Dan E. Meyer, Jennifer I. Luebke

**Affiliations:** 1 Department of Anatomy and Neurobiology, Boston University Chobanian and Avedisian School of Medicine, Boston, MA 02118 USA; 2 Center for Systems Neuroscience, Boston University, Boston, MA 02215 USA; 3 Department of Mathematics, Franklin and Marshall College, Lancaster, PA 17604 USA; 4 Yale School of Medicine, 333 Cedar St, New Haven, CT 06510 USA; 5 GE HealthCare Technology and Innovation Center, Niskayuna, NY 12309 USA; 6 Nash Family Department of Neuroscience, Friedman Brain Institute, and Center for Discovery and Innovation, Icahn School of Medicine at Mount Sinai, New York, NY 10019 USA

**Keywords:** Cortical layers, Rhesus monkey, Cellular, Vascular, Fibrillar, Immunofluorescence

## Abstract

In this novel large-scale multiplexed immunofluorescence study we comprehensively characterized and compared layer-specific proteomic features within regions of interest of the widely divergent dorsolateral prefrontal cortex (A46) and primary visual cortex (A17) of adult rhesus monkeys. Twenty-eight markers were imaged in rounds of sequential staining, and their spatial distribution precisely quantified within gray matter layers and superficial white matter. Cells were classified as neurons, astrocytes, oligodendrocytes, microglia, or endothelial cells. The distribution of fibers and blood vessels were assessed by quantification of staining intensity across regions of interest. This method revealed multivariate similarities and differences between layers and areas. Protein expression in neurons was the strongest determinant of both laminar and regional differences, whereas protein expression in glia was more important for intra-areal laminar distinctions. Among specific results, we observed a lower glia-to-neuron ratio in A17 than in A46 and the pan-neuronal markers HuD and NeuN were differentially distributed in both brain areas with a lower intensity of NeuN in layers 4 and 5 of A17 compared to A46 and other A17 layers. Astrocytes and oligodendrocytes exhibited distinct marker-specific laminar distributions that differed between regions; notably, there was a high proportion of ALDH1L1-expressing astrocytes and of oligodendrocyte markers in layer 4 of A17. The many nuanced differences in protein expression between layers and regions observed here highlight the need for direct assessment of proteins, in addition to RNA expression, and set the stage for future protein-focused studies of these and other brain regions in normal and pathological conditions.

**Supplementary Information:**

The online version contains supplementary material available at 10.1007/s00429-024-02819-y.

## Introduction

A major goal of systems neuroscience is to gain a detailed and comprehensive understanding of how neocortical areas in the primate brain mediate diverse sensory, motor, and cognitive functions. The neocortex can be described as a relatively uniform-appearing gray matter sheet about 2 mm-thick in the rhesus monkey, by convention considered to have six layers of differentially distributed neurons, glial cells, fibers, and vasculature. The quantitative parameters and spatial distribution of these features, however, have so far been investigated at a rather macroscopic scale, due to technical limitations. This is nonetheless sufficient to indicate that functionally distinct cortical areas differ in several striking and many nuanced ways with regard to their intrinsic cellular and fibrillar architecture, connectivity, and topographic organizational features. Recent improvements in technical resolution and throughput promise significant advances in our understanding of the underpinnings of functional specificity in the neocortex.

The identification and differentiation of cortical areas by histological criteria has a long history. The Brodmann parcellation scheme (Brodmann 1905, 1909), which defined 52 separate cortical areas based on cytoarchitecture—the size, density, and distribution of neurons across the 6 layers—was influential and Brodmann’s area numbers remain in widespread use. Later studies using a combination of cytoarchitecture, myeloarchitecture (distinctive patterns of myelinated axons) as well as cholinesterase histochemistry have proposed a range of different parcellation schemes for the monkey cortex ranging from 91 (Markov et al. 2014) to 161 (Paxinos et al. 2000) distinct areas (for review, see Van Essen and Glasser 2018).

Neocortical areas have more recently been differentiated based on additional features such as laminar organization and density of neurotransmitter receptors. These demonstrate area-specific layering patterns across the macaque cerebral cortex that do not necessarily coincide with cyto- or myeloarchitectural laminar boundaries (Caspers et al. 2015; Palomero-Gallagher and Zilles 2019; Rapan et al. 2022, 2023; Froudist-Walsh et al. 2023). Indeed, the laminar profiles of receptor densities are distributed as gradients in an approximately caudal to rostral manner from sensory to high-order association cortices.

Significant new cellular subtype identification criteria at the molecular and cellular levels derive from major technological advances in genomics and in microscopy (Zhang et al. 2022; Choe et al. 2023; Jung and Kim 2023; Piwecka et al. 2023; Xing et al. 2023; Tian et al. 2023; Balaram et al. 2023). Single-nucleus RNA sequencing (sNucSeq) and spatial transcriptomics, by enabling high-resolution characterization of gene expression in individual cells, have revealed dozens of neuron types that can be clustered based on their transcriptomic properties in diverse brain layers and areas in mice, monkeys and humans (Tasic et al. 2018; Bakken et al. 2021; Berg et al. 2021; Khrameeva et al. 2020; Krienen et al. 2020; Lei et al. 2022; Lein et al. 2017; Yao et al. 2021; Zhu et al. 2018; Jorstad et al. 2023; Maynard et al. 2021; Chiou et al. 2023)*.* Jorstad et al. (2023) employed deep sNucSeq in eight human cortical regions spanning from primary sensory to association cortices, which comprised consistently 24 broad cell classes based on distinctive RNA expression. A systematic comparison of sensory and association cortices revealed a marked area-specific difference in relative proportions of excitatory neuron subclasses and further reported distinct laminar distribution patterns of both astrocytes and oligodendrocytes in the different cortical areas. Notably, the primary visual cortex was found to differ markedly from all other brain areas with regard to transcriptomic features, ratios of excitatory to inhibitory neurons, diversity of layer 4 excitatory neuron types and specialized inhibitory neurons (Jorstad et al. 2023). These approaches provide important information on the genetic diversity of cells in the neocortex but are only predictive of potential expressed protein phenotypes of these cells.

Here we used an iterative multiplexed immunofluorescence (MxIF) approach to compare the laminar distributions, intensities and colocalization of a comprehensive panel of 28 different cellular, fibrillar and vascular proteins in the same individual sections prepared from the primary visual cortex (V1, area 17) and dorsolateral prefrontal cortex (DLPFC, area 46) of middle-aged rhesus monkeys. These areas lie at two poles of the telencephalon and differ extensively in their laminar cyto- and myeloarchitectural patterns. V1 in the macaque monkey has a prominent and complex layer 4 with at least 4 sublayers, and functionally V1 is a prototypical primary sensory cortex. In contrast, DLPFC has a more homogeneous layer 4 and is representative of association cortex involved in cognitive function. These two areas are also differentially vulnerable to the effects of normal aging and Alzheimer’s disease in humans, with the DLPFC exhibiting earlier and more severe vulnerability compared to V1 (Braak and Braak 1991; Hof and Morrison 1990; Hof et al. 1990; Grothe et al. 2018; Mrdjen et al. 2019).

We sought to determine whether the expression of a broad range of proteins would differentiate layers within and between these two highly distinctive neocortical areas. These proteins included 11 cellular, 4 fibrillar and 3 vascular markers, as well as 10 markers of oxidative stress, misfolded proteins, and other intracellular proteins. The numerous identified area-specific laminar proteomic profiles reveal relationships across multiple cell types and neuropil markers, setting the stage for future investigations of cellular-molecular interactions in diverse regions in the normal young, aged and pathological primate brain.

## Materials and methods

### Subjects and tissue preparation

**Fig. 1:**
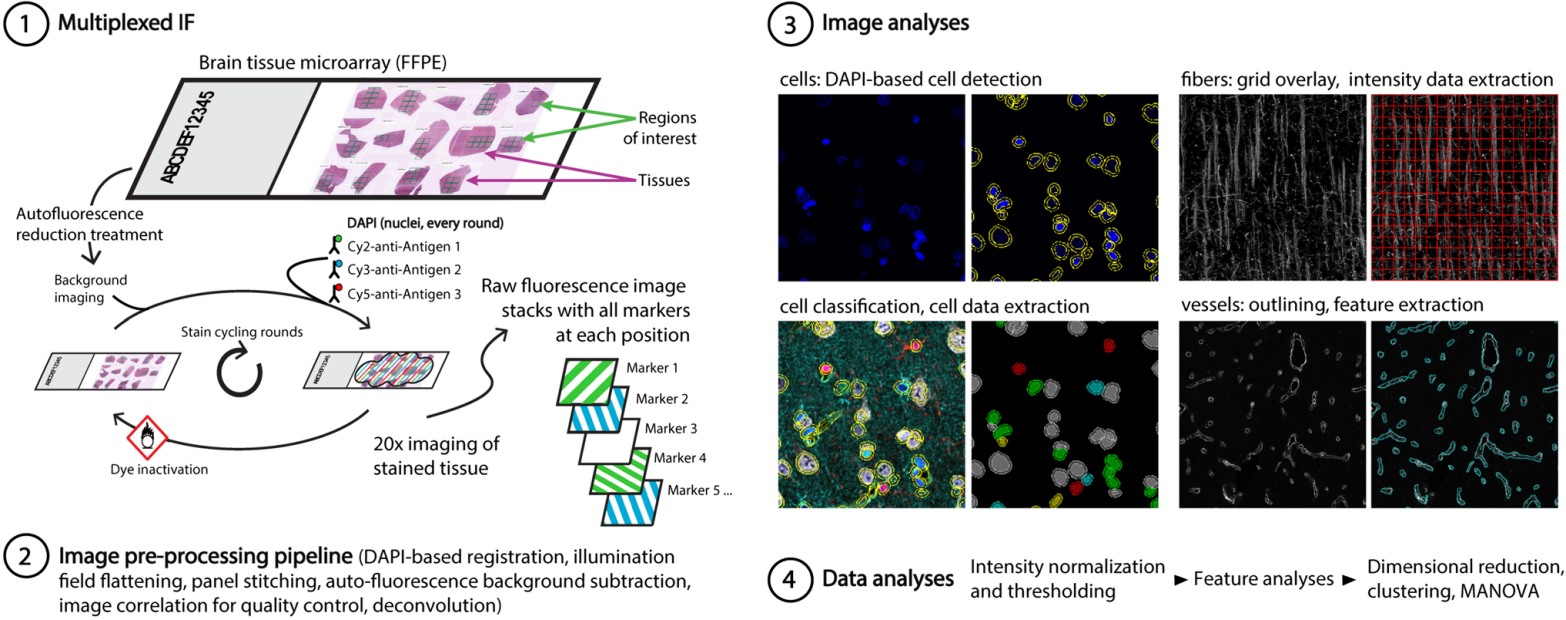
Overall experimental design for this study. Formalin fixed paraffin embedded (FFPE) tissues as TMAs on microscope slides undergo sequential rounds of MxIF, each staining up to three markers (1). Resulting epifluorescence images are pre-processed (2), and then analyzed (3). Resulting data are intensity normalized and thresholded, then features and intensity data are extracted and analyzed with a variety of statistical approaches (4)

Brain tissue was obtained from seven middle-aged rhesus monkeys (*Macaca mulatta*, Table 1) with complete health records that were acquired from national primate research facilities and from private vendors and were part of our larger studies of normal brain aging. All procedures were approved by the Boston University Institutional Animal Care and Use Committee and were conducted in strict accordance with the Guide for the Care and Use of Laboratory Animals (National Research Council 2011). Monkeys were sedated with ketamine hydrochloride (10 mg/kg), subsequently anesthetized with sodium pentobarbital (15 mg/kg, to effect) and then perfused through the ascending aorta with ice-cold Krebs–Henseleit buffer, which contained, in mM: 6.4 Na_2_HPO_4_, 1.4 Na_2_PO_4_, 137 NaCl, 2.7 KCl, 5 glucose, 0.3 CaCl_2_, and 1 MgCl_2_, pH 7.4 (Sigma-Aldrich, St. Louis MO USA). Roughly cubic brain tissue blocks with edges about 10 mm in size were taken from the frontal and occipital cortices. The frontal region of interest was in Brodmann area 46 (A46) located in the caudal half of the ventral bank of the principal sulcus within the DLPFC (Barbas and Pandya 1989). The occipital primary visual cortex region of interest was within the lateral region of V1, Brodmann area 17 (A17; Rockland and Pandya 1979). Blocks were immersion-fixed immediately in 4% paraformaldehyde for 48 h at 4 °C. Fixed tissue blocks were then further blocked into 3-mm-thick blocks, placed in cassettes, and embedded in paraffin according to the following protocol: 6 steps of graded (70%, 80%, 95%, 95%, 100%, 100%) ethanol at 37 °C for 10 min each; 2 steps of xylene at 37 °C for 10 min each; 3 steps of paraffin wax at 57 °C for 10 min each. These blocks were then used as donors in the construction of tissue microarrays (TMAs, Fig. 1), which were made using 4.5-mm punches of the donor blocks that were arrayed into a 3-by-5 spot format with pial surface oriented upwards (Pantomics Inc., Richmond, CA). The resulting TMAs were sectioned at 5 µm thickness onto charged, 75 × 25-mm glass microscope slides (SuperFrost™ Plus, Thermo Fisher Scientific, Waltham, MA). One tissue section per area/subject was used for all subsequent analyses. The Cell DIVE™ MxIF procedure including staining of sections, image acquisition and pre-processing pipeline, image analysis and data analyses is shown graphically in Fig. 1 and described in detail below.

**Table 1:** Subjects

Subject	Age	Sex	A46	A17	DRST-S	DRST-O
AM303	15.3	Male	√	√	2.44	3.93
AM311c	20.3	Male	√	–	3.19	3.15
AM340	17.6	Female	√	√	2.12	2.76
AM342c	19.2	Female	√	√	2.94	2.73
AM347c	14.6	Female	√	–	2.45	3.3
AM350c	17.3	Female	√	√	3.92	2.52
AM353c	14.5	Male	–	√	2.68	4.01

*DRST* delayed recognition span task, *S* spatial, *O* object

### Slide preparation

Formalin-fixed, paraffin-embedded (FFPE) tissue sample slides were deparaffinized and subjected to antigen retrieval as previously described in Gerdes et al. (2013), with minor modifications. In brief, sample slides were baked at 60 °C overnight, deparaffinized with xylene, and rehydrated by 2 × 5-min aqueous washes with decreasing ethanol concentrations (xylene, 100%, 95%, 70%, 50%, ethanol, and 1 × PBS), and then permeabilized with a 10-min incubation in 0.3% TritonX-100 in phosphate buffered saline (PBS). Next, the slides underwent a two-step antigen retrieval treatment using 10 mM citrate buffer (Vector Labs, Newark, CA) at pH 6.0 and TRIS buffer (10 mM TRIS, 1 mM EDTA, 0.5 mM Tween20) at pH 8.5. This was accomplished in a sealed pressure vessel at nominal 4psi and ramped to 110 °C. Next, to inhibit nonspecific binding, slides were incubated overnight at 4 °C with 10% (wt/vol) donkey serum, 3% (wt/vol) bovine serum albumin (BSA) in PBS. To reduce the background fluorescence of neuronal tissue, slides were exposed to a multi-array LED illumination (mixed LEDs comprising 660 nm and 610 nm red, 460 nm blue, and 6500K white; AgroMax OTD84 LED Grow Light, HTG Supply, Callery, PA) for 72 h at 4 °C while in 0.02% sodium azide in PBS. Slides were then incubated with 1 µg/ml (wt/vol) 4′,6-diamidino-2-phenylindole (DAPI) and coverslipped with mounting medium consisting of 80% (vol/vol) glycerol in 1 M TRIS.

### Antibody labeling and validation

A panel of markers for cellular, fibrillar and vascular components of the neuropil were selected as optimal for comprehensive assessment of protein constituents of neocortical gray and white matter (Table 2). A set of target antibodies were evaluated in a series of tests to select an optimal antibody for MxIF imaging using positive control FFPE tissue as previously published (McDonough et al. 2020). Antibodies were selected based on staining specificity and sensitivity in indirect immunofluorescence and their retention of specificity after the sample tissue was exposed to dye inactivation chemistry. Specificity was assessed qualitatively by visual inspection of expected localization patterns. Positive control tissue slides were prepared as described above and incubated with the target antibody in 3% (wt/vol) BSA in PBS for 1 h at room temperature. For secondary detection, slides were washed with PBS and a species-specific, dye-labelled IgG, at a working concentration of 5 µg/ml (wt/vol) or 1:250 in 3% BSA in PBS for 1 h at room temperature. After another PBS wash, the test slides were coverslipped with mounting medium as above. To identify the best antibodies for each marker, up to three different antibodies were tested; optimal antibodies selected for the study are specified in Supplementary Table 1. Once selected, the primary antibody was either (i) directly conjugated to a cyanine (Cy) dye as previously published (Sood et al. 2021), (ii) dye labelled with a Zenon™ kit (Thermo Fisher Scientific, Waltham, MA) following manufacturer’s protocol and used within 30 min of labelling, or iii) purchased as dye-conjugated (Suppl. Table 1). The dye-labelled antibodies were then stained as above and evaluated for direct immunofluorescence. As an additional element of validation, the potential loss of antigens targeted following dye inactivation were evaluated using control slides that were repeatedly dye inactivated to mimic 1 ×, 5 ×, and 10 × rounds of multiplexed immunofluorescence, then stained for comparison to a control slide that had not received dye inactivation treatment. The specificity and sensitivity of each conjugate was evaluated, using at least two antibody dilutions, in comparison to the unconjugated primary antibody. If direct immunofluorescence failed, the antibody was included in the study as an indirect stain.

**Table 2:**
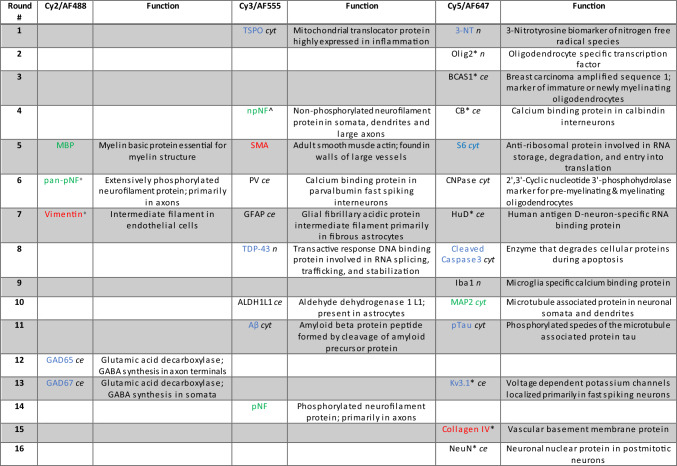
Markers assessed in this study in consecutive staining rounds (markers indicated in each row/round) on the same tissue sections. Color coding: cellular, , ,

Abbreviations indicating cellular location of protein *ce* = cellular (nuclear + cytoplasmic); *cyt* = cytoplasmic; *n* = nuclear; Cy = cyanine; +  = AF488 dye; ^ = AF555 dye; * = AF647 dye (See Suppl. Table 1)

### Multiplexed immunofluorescence imaging

Procedures for dye-deactivation-based MxIF have been previously published (Gerdes et al. 2013; McDonough et al. 2020). Prior to staining, each slide was imaged to acquire unstained tissue background and generate virtual H&E images (vH&E, generated using DAPI stain and Cy3 tissue autofluorescence images) for pathology review and selection of regions of interest. Following image acquisitions, coverslips were removed by inverting the slide and incubating in PBS at room temperature, allowing the coverslip to gently detach from the slide over time. Once ready for staining, antibodies were diluted in a 3% (vol/vol) BSA solution in PBS to their respective optimized concentrations (typical range 0.1–10 μg/ml, see “working concentration” in Suppl. Table 1) and applied for 1 h at room temperature using autostainer (Leica BOND-MAX™, Leica Biosystems, Deer Park, IL). Samples were then washed with 1 × Bond wash (Leica Biosystems) in three exchanges, 5 min each. In the case of secondary antibody detection, samples were incubated with a primary antibody species-specific secondary donkey IgG conjugated to either Cy3 or Cy5, for 1 h at room temperature using an autostainer. Slides were then washed with 1 × Bond wash as described above. When slides were ready for imaging, they were rinsed with PBS, then mounted with coverslips using mounting medium described above. Slides were then imaged via an IN Cell Analyzer 2200 (Leica Microsystems, Wetzlar, Germany) using a 10 × objective to set field focus and alignment, then 20 × for image capture. Exposure times were set manually after evaluating multiple fields of view prior to acquisition. After image acquisition from a round of staining, coverslips were removed as above and slides underwent dye inactivation by incubating them in an alkaline solution containing H_2_O_2_ for 15 min at room temperature, then washing with PBS. The slides were then coverslipped and imaged to acquire a new background image post dye inactivation. After re-imaging, coverslips were removed again as described above and restained with another round of antibodies via Leica Bond stainer as described above. Sample slides were imaged again for target staining. The cycle of staining, imaging, dye inactivation, imaging was repeated through all targets. Sample slides underwent iterative staining and imaging of 28 markers across 15 staining rounds (Table 2).

### Image preparation

**Fig. 2:**
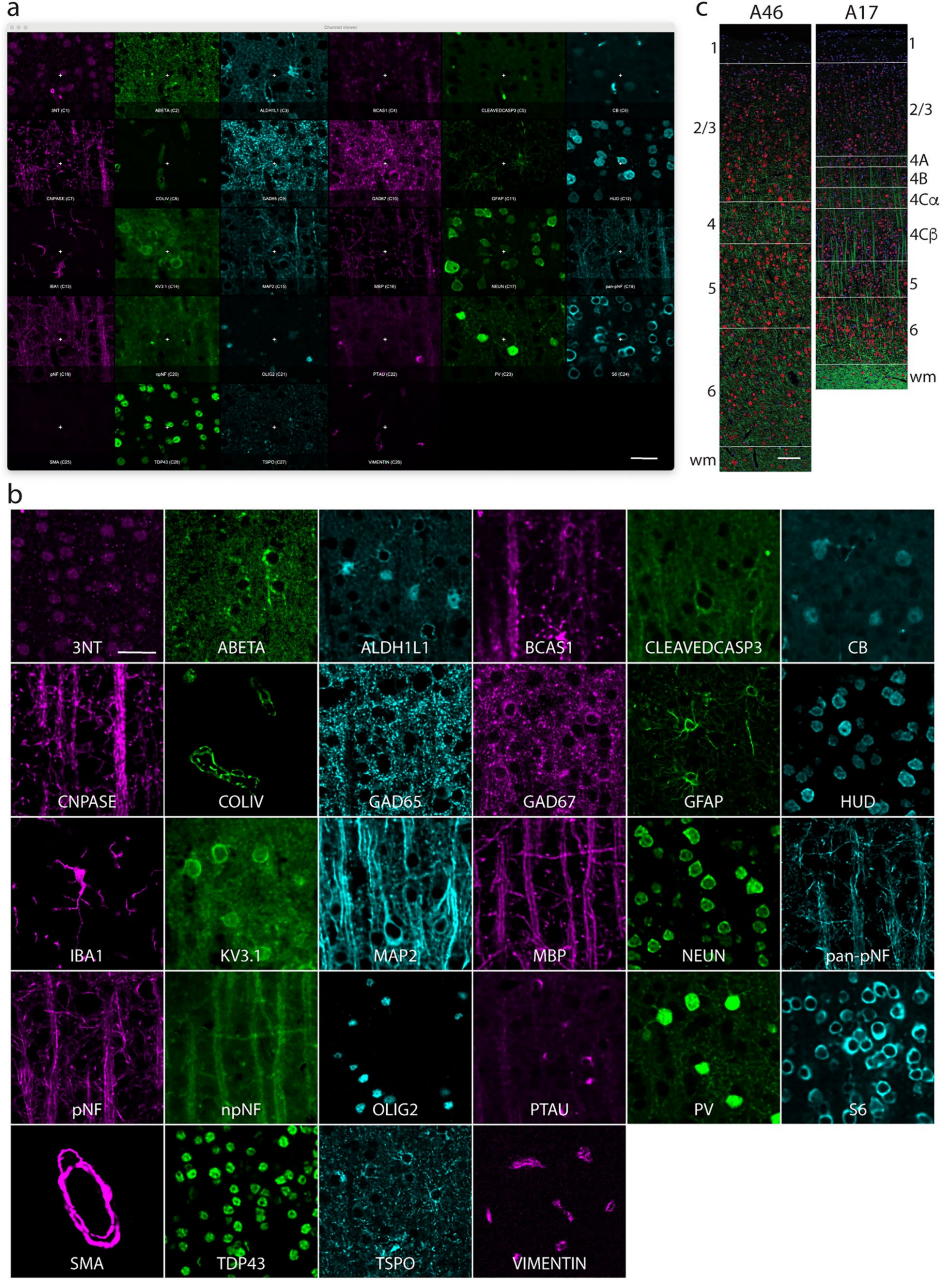
Markers and tissues analyzed in this study. **a** Photomicrographs of the 28 markers in the identical field of view (marker colors are randomly assigned). **b** Photomicrographs showing representative staining for each marker (different fields of view) **c)** 400-µm wide ROIs from A46 and A17. All images are from a 17.3-year-old female monkey. Blue = DAPI; red = HuD; green = MBP; White lines delineate laminar boundaries. Scale bars: **a**, **b** = 25 µm; **c** = 100 µm

Background-subtracted, epifluorescence images were rotated to orient the pial surface horizontally and cropped to regions of interest (ROIs) 400-µm wide and extending from the pial surface through the white matter. ROIs were chosen to avoid tears or artifacts present in the tissue. Cropped images were then deconvolved using AutoQuant X3 software (Meyer Instruments). Multi-channel OME-TIF files were created for each individual tissue section using the deconvolved images. Laminar boundaries were delineated based on the literature (Barbas and Pandya 1989; Rockland and Pandya 1979); these laminar boundaries are indicated by white lines in Fig. 2b (Suppl. Table 2). In this study we did not discriminate between layers 2 and 3 and present these laminar data as layer 2/3. For A17, layer 4 was further subdivided into 4 sublayers (Lund et al. 1994), with 4A, 4B, 4Cα, and 4Cβ limits based on an approximation of the percentage of the total depth of the layer belonging to each sublayer—10%, 20%, 20% and 50%, respectively. Areas for all cortical layers were calculated using the individual layer limits except for layer 1. Due to the variability of the pial surface curvature, layer 1 area was calculated in QuPath (v0.3.2; Bankhead et al. 2017) by creating an annotation that outlined this layer only and extracting its area value. For all images, the total sampled depth is from the pial surface to 100 µm into the white matter, except for one section that did not have white matter to be sampled.

### Cellular analyses

Using the image analysis open-source software QuPath, images were added to a single project to allow for batch processing. A custom script that implements the software’s Cell Detection function with uniform parameters and segments objects based on DAPI staining was run for the whole project via batch processing. The cellular detection parameters included a cell expansion of 2 µm from the nucleus, allowing us to gather data from three compartments defined by QuPath as “Cell” (inclusive of “Nucleus” and “Cytoplasm”), “Cytoplasm” (excludes “Nucleus”) and “Nucleus” (excludes “Cytoplasm”). Having a conservative expansion of 2 µm ensures that the data collected is indeed from the cell corresponding to a given DAPI object (Fig. 1). Once all DAPI objects were segmented, these were manually classified into different categories: “Neuron”, “Astrocyte”, “Microglia”, “Oligodendrocyte”, “Endothelial Cell”, “Other”, or “Multiple”. Objects were classified based on the presence of cell type-specific markers: HuD and NeuN for neurons; ALDH1L1 and GFAP for astrocytes; Iba1 for microglia; Olig2 for oligodendrocytes (and BCAS1 and CNPase); and collagen IV and vimentin for endothelial cells. Objects that contained more than one cell type specific marker were classified as “Multiple” while those that did not appear to fall within any of the cell type classifications were classified as “Other”. Since these objects represented a small proportion of classified objects (~ 4% in both brain areas) they were excluded from subsequent analyses. The resulting dataset included location, shape, and all marker intensity data for all detections. Coordinates for each cell were then used to assign each cell to a cortical layer based on previously defined layer limits. Counts, densities, and relative proportions of the different cell types were calculated both within layers as well as in the sampled depth. Relative intensities of different pathological markers within the different cell types and neuron subgroups were also assessed.

### Analysis and thresholding of marker intensities

Since data on cell marker intensities are known to be log-normally distributed (Bagwell 2005; Lin et al. 2015, 2018, 2023), we took the natural logarithm of raw intensity values, after shifting all values by 1 to avoid the discontinuity of ln(0). We then applied a z-score transformation to all intensity values in each tissue section. These data, hereafter called mean grayscale intensity (MGI), were then combined for comparison across all tissue sections as a proxy for protein expression level. Some of the cellular markers are known to express primarily in certain cell types, which allowed us to assign threshold values of MGI for each marker to decide whether a given cell was considered as expressing that marker. We manually determined appropriate thresholds in the reference cell type (Suppl. Table 3). These thresholds in the reference cell type then identified corresponding threshold values of MGI: any cell with an MGI value above that threshold was considered positive for that marker; otherwise, the cell was negative for that marker (Suppl. Fig. 1)

### Marker colocalization analysis and MGI dimension reduction

We selected 21 markers for cell types and intracellular proteins (all markers except those for fibers, namely neurofilament markers and myelin, or vessels, namely vimentin, SMA, and collagen IV) and removed the lowest 0.2% of MGI values for each. Separately for each cell type (neurons, oligodendrocytes, astrocytes, and microglia) we performed a principal components analysis of the MGI for all 21 markers. In each case, we identified the principal components (PCs) with largest eigenvalues that collectively accounted for 80% of the variation in the data and examined the scores of each cell projected onto those PCs. For neurons, this was the first 8 PCs. A two-way ANOVA of these scores was performed, determining whether the scores projected onto each PC differed by Brain Area and Layer. Finally, as a top-down cluster analysis, we performed a multivariate analysis of variance (MANOVA) of MGI values to determine which brain area/layer combination differed significantly from the others.

### Blood vessel analyses

To analyze blood vessel properties, a new QuPath project was created that included the same multi-channel images used for the cellular analyses. Individual pixel classifiers were trained to recognize positive staining for vimentin, collagen IV, and smooth muscle actin (SMA). Using the collagen IV pixel classifier, we created objects based on positive pixel classification and manually edited them to ensure they captured the whole vessel to accurately obtain measurements such as area, perimeter, circularity, and area of positive pixels for each marker within the vessel objects.

### Fiber analyses

For analysis of fibrillar markers, images were added to a new QuPath project and a script was created to overlay a grid of 20 × 20 µm tiles on the image, starting at the pial surface and ending at the bottom of the image and generate intensity measurements for selected markers. Intensity measurements within each tile were used to sample the expression of the markers across the tissue section. The resulting data were exported and, for each individual image and marker, the mean intensity measures of the tiles were normalized by using a log + 1 transformation and then z-scoring the resulting values. For each image and marker, the transformed data for all tiles at each depth were averaged to obtain an average-at-depth measurement for all depths within a tissue section. All depths were then assigned to a layer based on individual image coordinates and normalized to a depth scale with the pia at 0 and the white matter line at 100 for all tissue sections.

### Statistical analyses

**Fig. 3:**
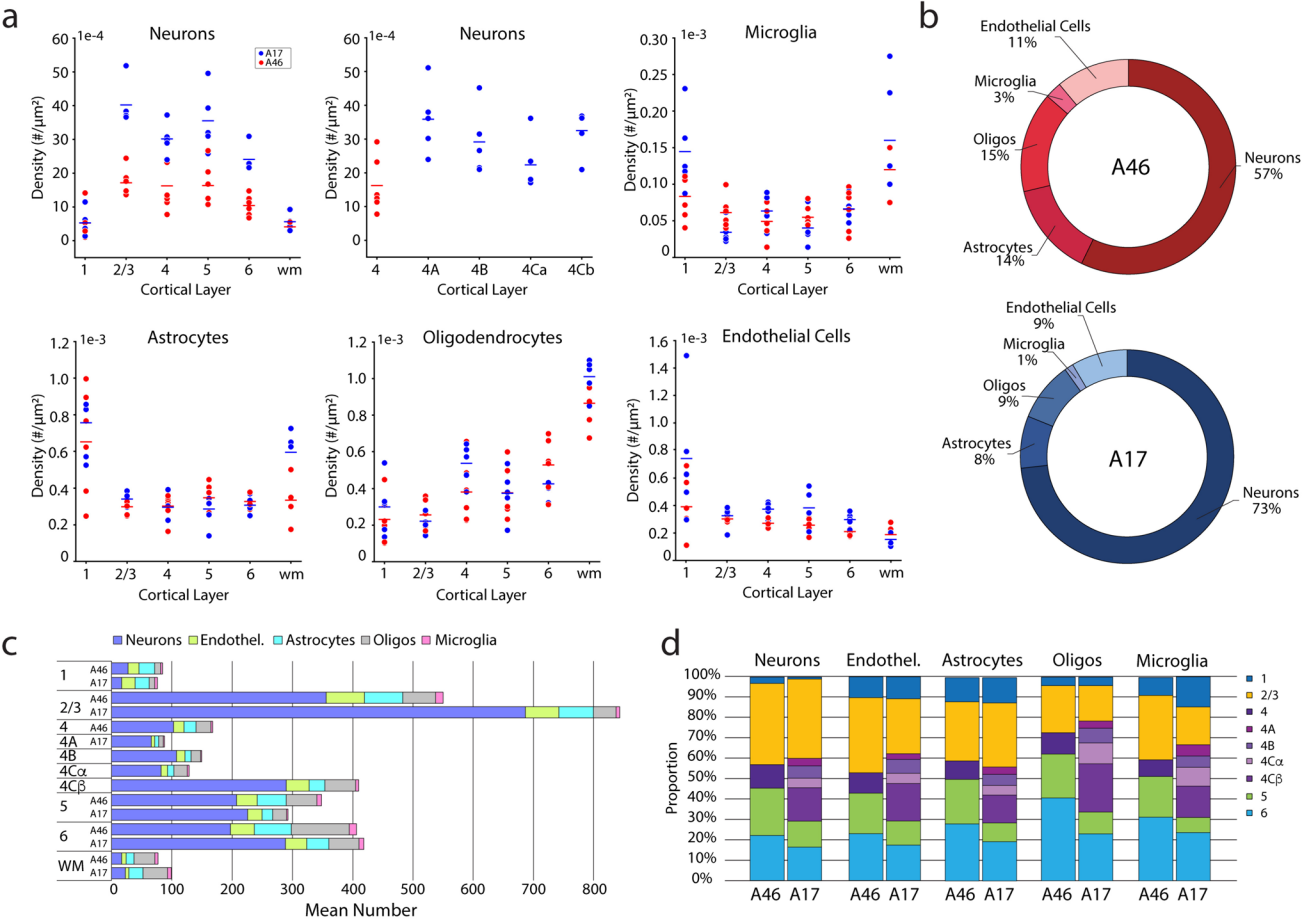
Density, number, and proportions of the five cell classes in A46 vs. A17. **a** Density of neurons across cortical layers and white matter and in A17 L4 sublayers (top left and middle). Each dot represents the mean value for one monkey. The density of microglia (top right) astrocytes (bottom left), oligodendrocytes (bottom middle) and endothelial cells (bottom right) are shown for each layer and WM. There were no differences in the density of these cells within A17 L4 sublayers so these data are not shown. **b** Ring graphs showing the relative overall proportions of the five cell types. **c** Graph showing the numbers of neurons, endothelial cells, astrocytes, and oligodendrocytes in each layer of A46 and A17. **d** Bar graphs showing the proportions of each of the five main cell types across the six cortical layers

**Fig. 4:**
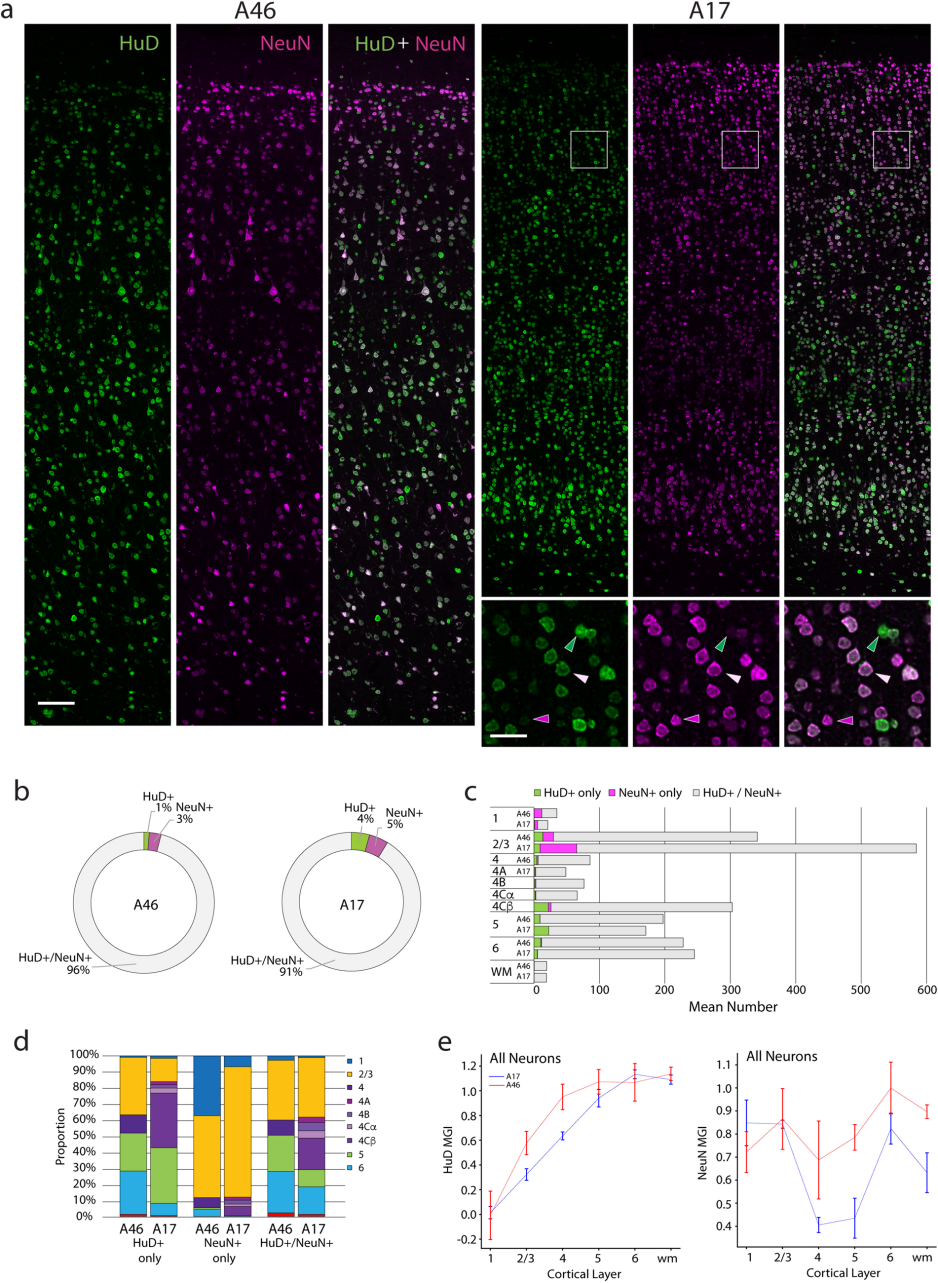
Number, proportions, and marker intensity of HuD+ and NeuN+ neurons in A46 vs. A17. **a** Photomicrographs showing HuD + (green), NeuN+ (magenta) and double-labeled (white) neurons in A46 and A17 (long panels) from a 17.3-year-old female monkey. Small panels are zoomed-in micrographs from A17 (white boxed area, rightmost panel). Scale bars: left, 100 µm; right, 25 µm **b** Ring graphs showing the relative overall proportions of neurons that were both HuD + /NeuN+ vs those that contained only HuD + or only NeuN+. **c** Graph showing the numbers of HuD + only, NeuN+ only and HuD + /NeuN+ neurons in each layer of A46 and A17. **d** Bar graphs showing the relative proportions of HuD+/NeuN+, HuD+ only and NeuN+ only neurons across laminae and in WM. **e** Mean grayscale intensity (MGI) of HuD (left) and of NeuN (right) in all neurons across cortical layers

**Fig. 5:**
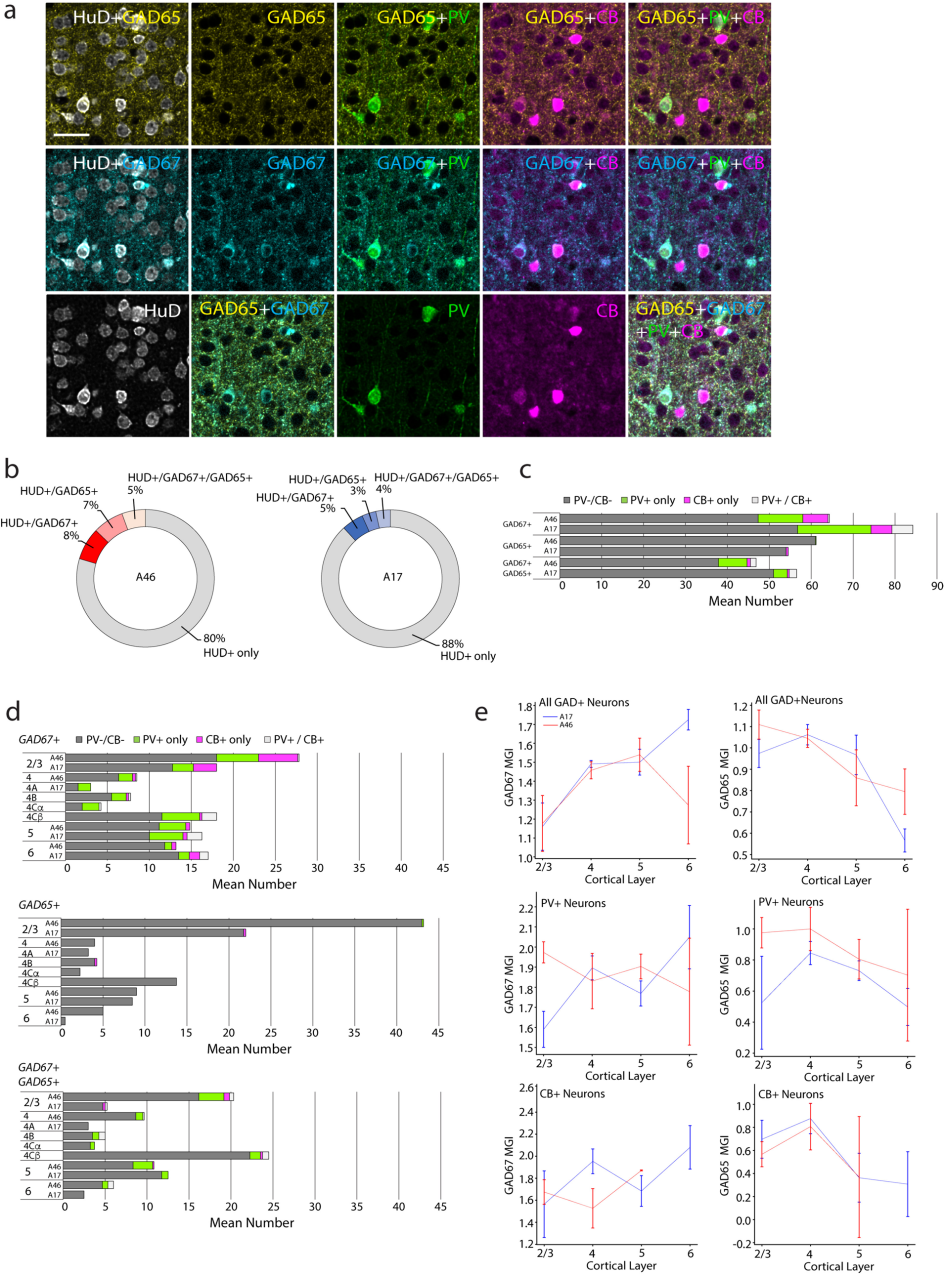
Number, proportions, and marker intensity of inhibitory neurons in A46 and A17. **a** Photomicrographs showing GAD65+ (yellow), GAD67+ (cyan), PV+ (green) and CB+ (magenta) in A17 of a 14.5-year-old male monkey. Scale bar: 25 µm. **b** Ring graphs showing the relative overall proportions of neurons that were positive for each of the 4 inhibitory neuron protein markers or double-labeled with two of these markers. **c** Graph showing the mean numbers of GAD65+  or GAD67+ neurons that were positive for PV, CB or both PV and CB in A46 and A17. **d** Graph showing the numbers of GAD67 + (top), GAD65+ (middle) or GAD65+/GAD67+ neurons that were positive for PV, CB or both PV and CB in each layer of A46 and A17. **e** Mean grayscale intensity (MGI) of GAD67 (left column) or GAD65 (right column) in all GAD+ neurons (top), PV+ (middle) and CB+ (bottom) neurons across cortical layers

**Fig. 6:**
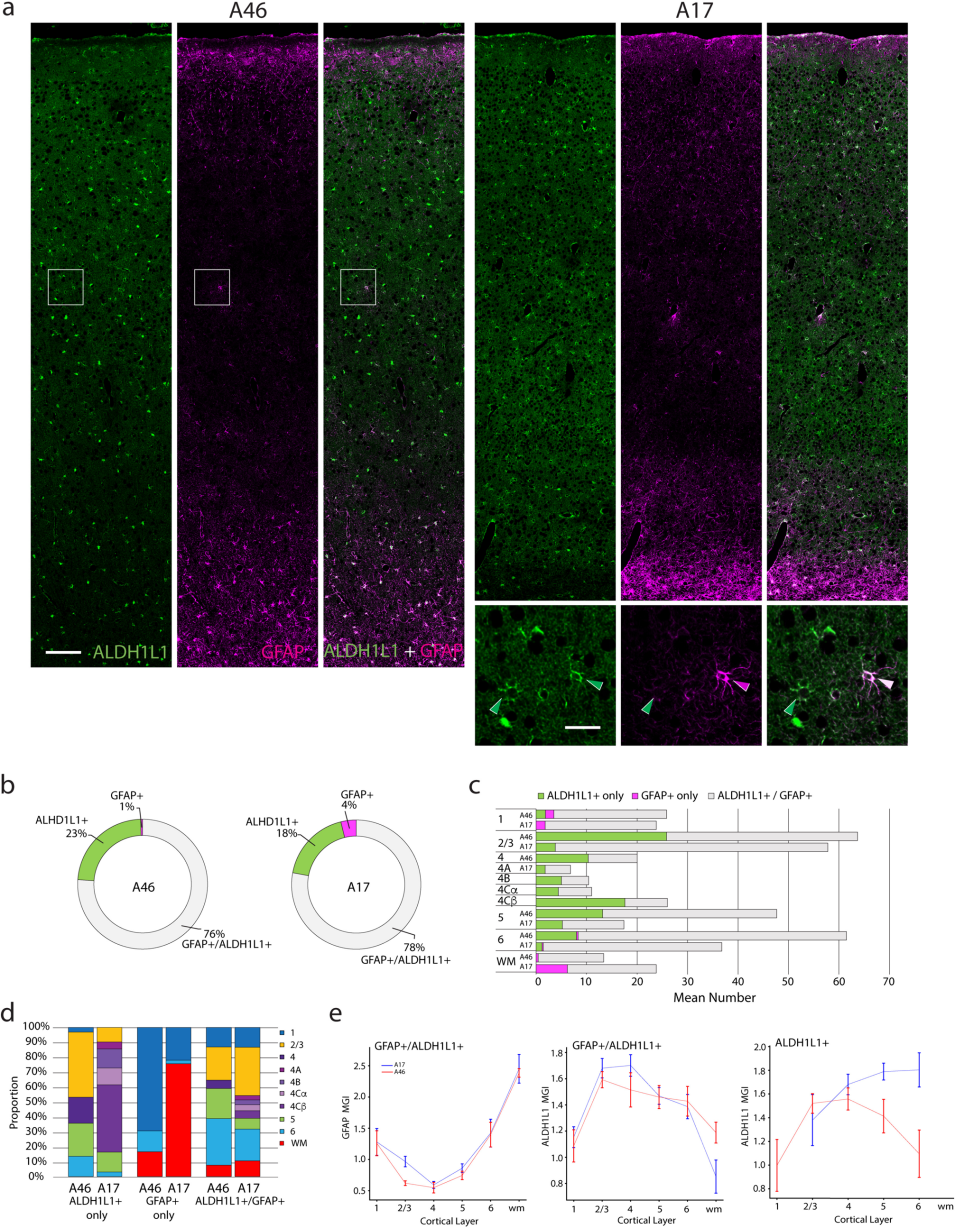
Number, proportions, and marker intensity of GFAP+ and ALDH1L1+ astrocytes in A46 vs. A17. **a** Photomicrographs showing ALDH1L1+ (green), GFAP+ (magenta) and double-labeled (white) neurons in A46 and A17 (long panels) from a 15.3-year-old male monkey. Small panels are zoomed micrographs from A46 (white boxed area left panels). Scale bars: left, 100 µm; right, 25 µm. **b** Ring graphs showing the relative overall proportions of neurons that were both GFAP+/ALDH1L1+ and those that stained only ALDH1L1 or only GFAP. **c** Graph showing the mean number of ALDH1L1+ only, GFAP+ only and GFAP+/ALDH1L1+ astrocytes in each layer of A46 and A17. **d** Bar graphs showing the relative proportions of GFAP+/ALDH1L1+, ALDH1L1+ only and GFAP+ only astrocytes across laminae and in the white matter. **e** Mean grayscale intensity (MGI) of GFAP in GFAP+/ALDH1L1+ (left) and of ALDH1L1 in ALDH1L1+ only (right) astrocytes

**Fig. 7:**
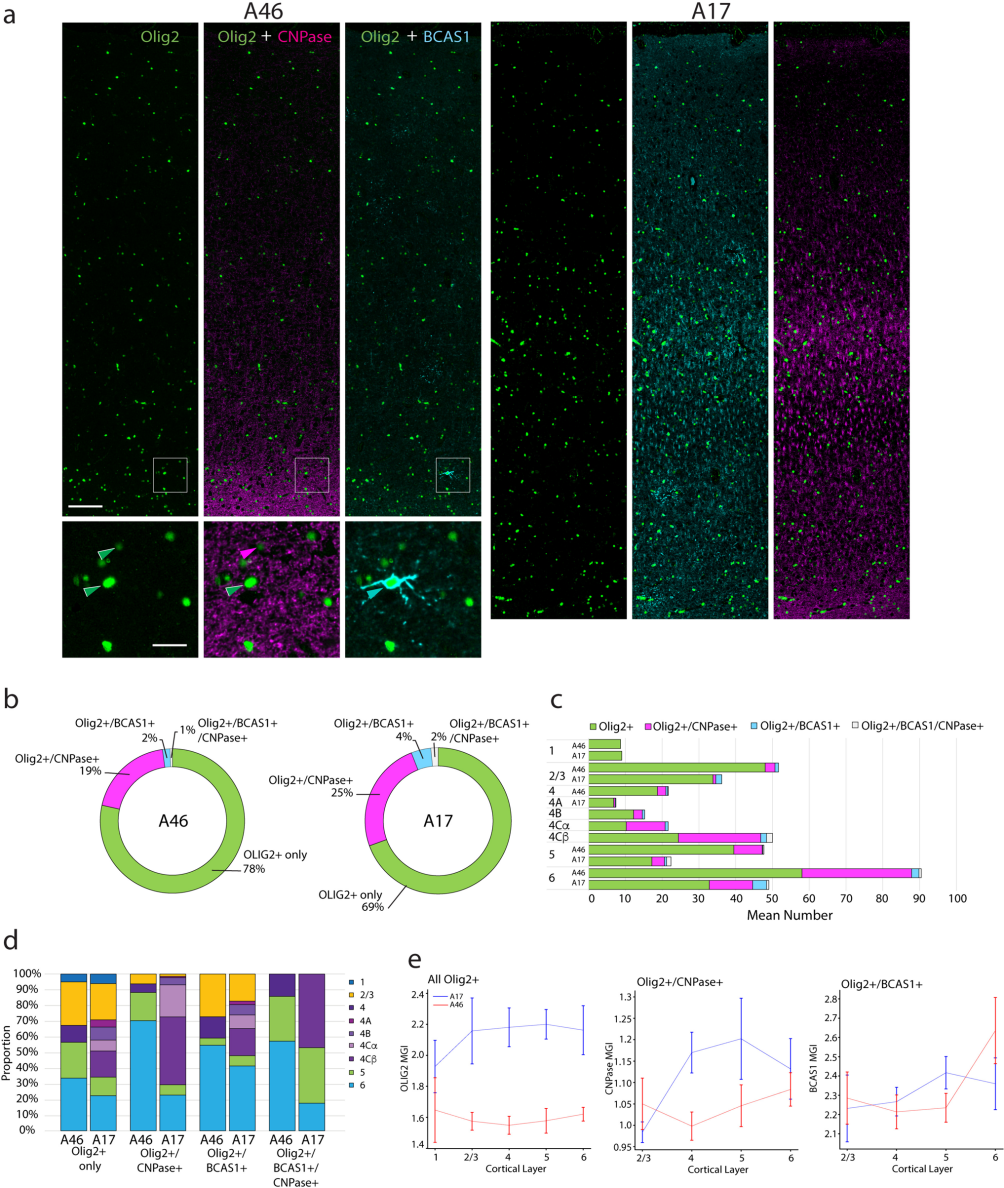
Number, proportions, and marker intensity of Olig2+, CNPase+ and BCAS1+ oligodendrocytes in A46 vs. A17. **a** Photomicrographs showing Olig2+ (green), CNPase+ (magenta) and BCAS1+ oligodendrocytes in A46 and A17 (long panels) from a 15.3-year-old male monkey. Small panels are zoomed micrographs from A46 (white boxed area top panels). Scale bars: left, 100 µm; bottom, 25 µm. **b** Ring graphs showing the relative overall proportions of oligodendrocytes that were Olig2+ only vs Olig2+/CNPase+, Olig2+/BCAS1+ or contained all 3 oligodendrocyte markers. **c** Graph showing the mean number of Olig2+ only, Olig2+/CNPase+, Olig2+/BCAS1+ and Olig2+/CNPASE+/BCAS1+ oligodendrocytes in each layer of A46 and A17. **d** Bar graphs showing the relative proportions of oligodendrocytes staining for the 3 markers across laminae in A46 and A17. **e** Mean grayscale intensity (MGI) of Olig2 (left), CNPase (middle) and of BCAS1 (right) in all Olig2+, Olig2+/CNPase+, Olig2+/BCAS1+ oligodendrocytes respectively

**Fig. 8:**
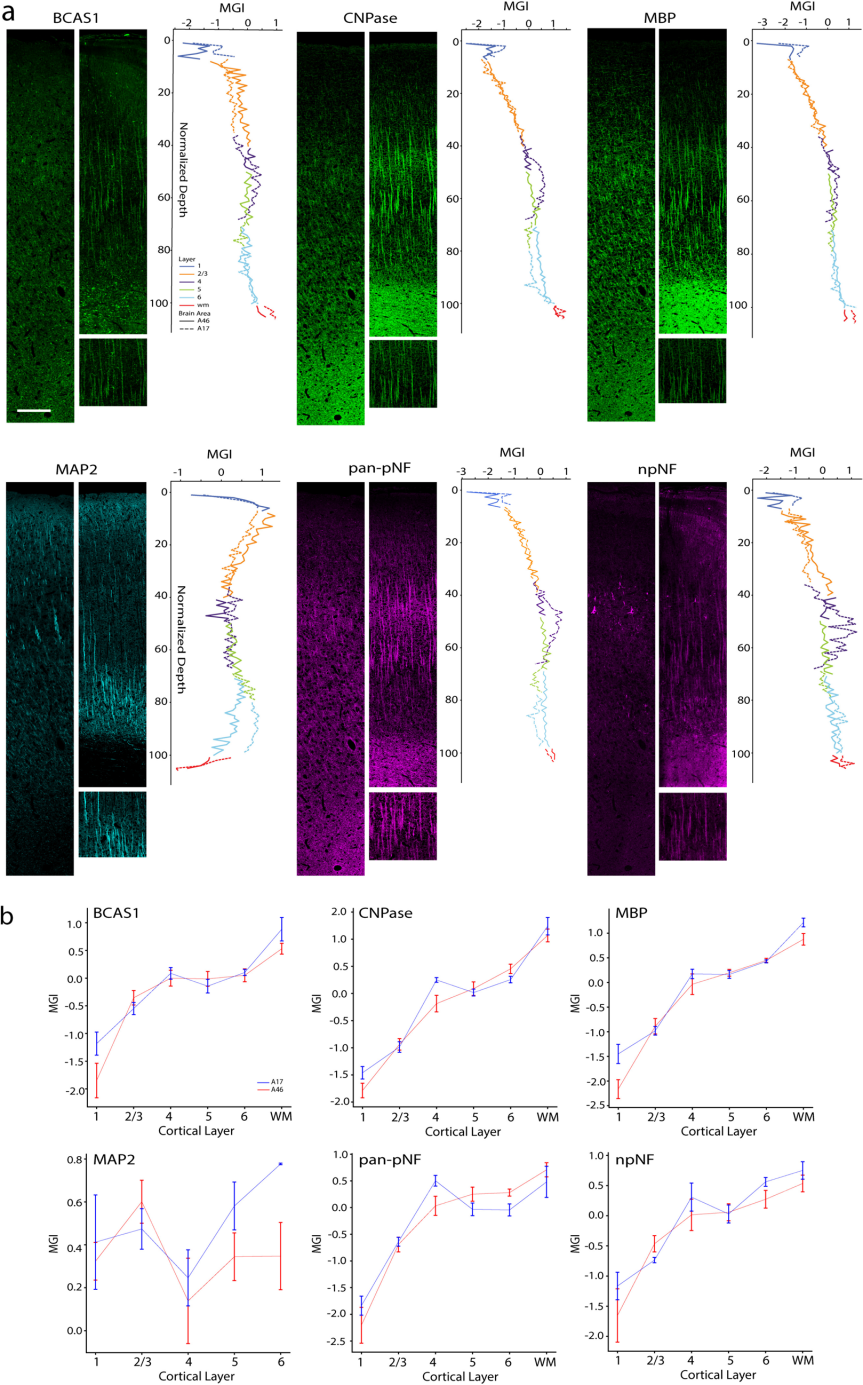
Intensity of 6 markers for fibrillar components of the neuropil in A46 vs. A17. **a** left—Photomicrographs showing the 6 fibrillar markers—BCAS1, CNPase, MBP, MAP2, pan-pNF and npNF in A46 and A17 (long panels) from a 17.3-year-old female monkey. Scale bar: 100 µm. Zoomed-in images of fibers from the white boxed area in A) A17. Right—Line graphs showing (from top to bottom) the mean grayscale intensities (MGIs) of BCAS1, CNPase, MBP, MAP2, pan-pNF and npNF as a function of normalized depth in the two brain areas. **b** Mean grayscale intensity (MGI) of each of the 6 markers across cortical layers in A46 and A17

**Fig. 9:**
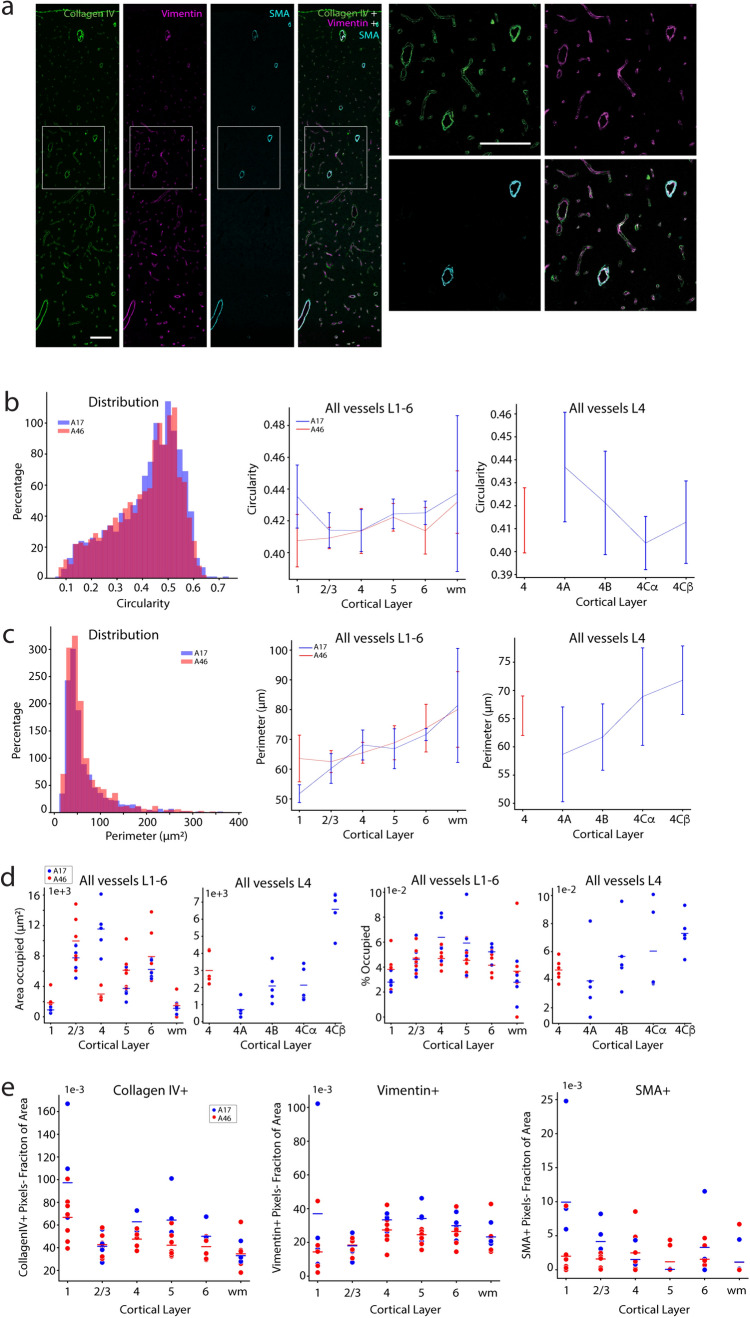
Characteristics of vascular markers in A46 vs. A17. **a** Photomicrographs showing the 3 vascular markers -collagen IV, vimentin and SMA- in A46 and A17 (long panels) and at a higher zoom from the A46 from a 17.6-year-old female monkey. Scale bars: 100 µm. **b** Left: Distribution of all (collagen IV+) vessels circularity in A46 vs A17. Middle: Mean circularity of vessels across layers. Right: mean circularity of vessels in A46 L4 and in the sublayers of A17 L4. **c** Left: histogram of vessel perimeters. Middle: line graph showing the mean perimeter of vessels across layers. Right: mean perimeter of vessels in A46 L4 and in the sublayers of A17 L4. **d** Area occupied by collagen IV+ vessels across layers (left two panels) and fraction of each layer occupied by vessels (right two panels). **e** Vertical dot plot graphs demonstrating the fraction of each layer and WM occupied by pixels positive for collagen IV (left), vimentin (middle) and SMA (right)

**Fig. 10:**
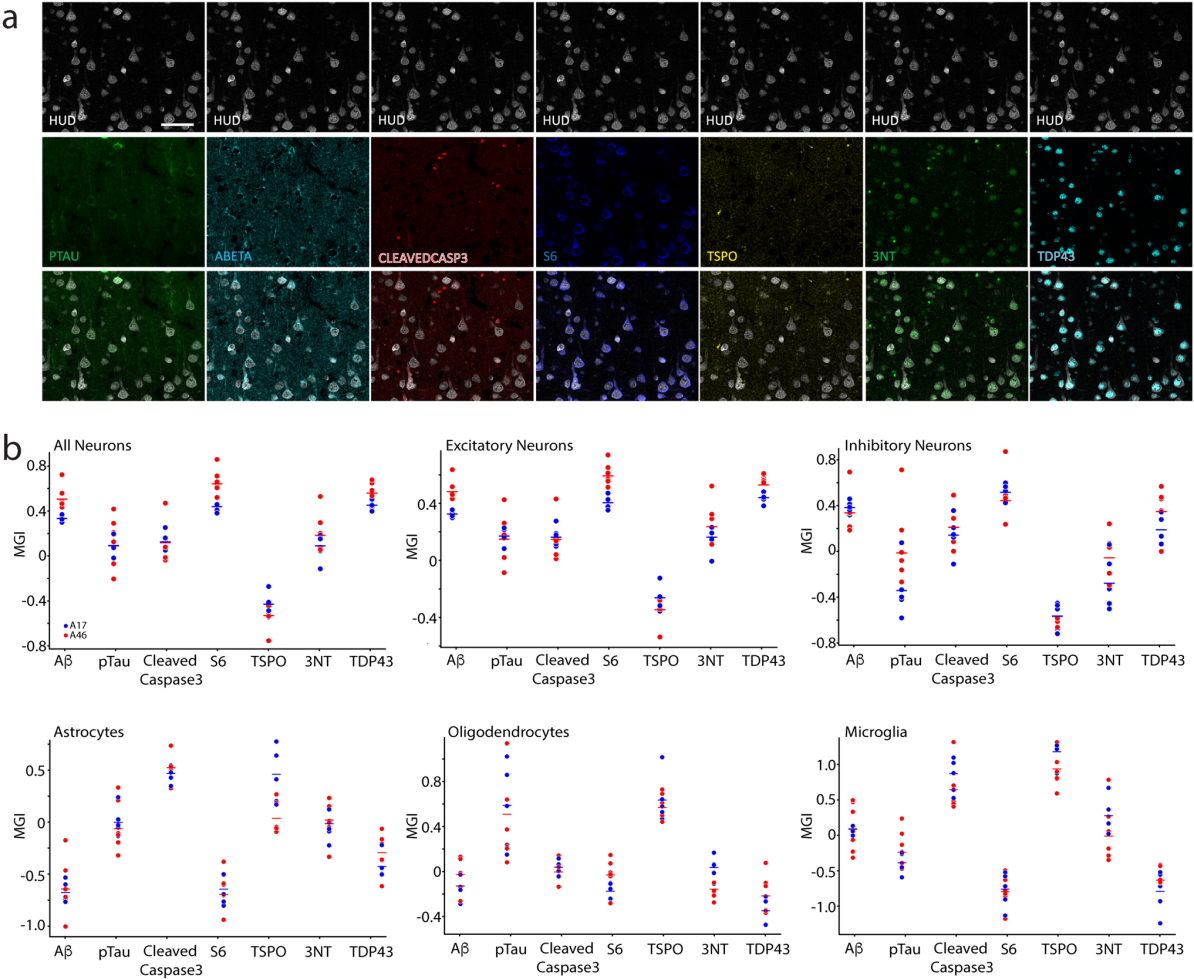
Oxidative stress and misfolded protein markers in A46 vs. A17 cell types. **a** Photomicrographs showing the presence of 7 different markers of oxidative stress or pathology in HuD+ neurons. Scale bar: 25 µm. **b** Vertical dot plots showing the mean grayscale intensities (MGIs) of the 7 markers—Aβ, pTau, Cleaved Caspase3, S6, TSPO, 3NT and TDP43- in top: all (NeuN+ and/or HuD +) neurons, excitatory (GAD67-) neurons and inhibitory (GAD67+) neurons; bottom: astrocytes (ALDH1L1+ and/or GFAP+), oligodendrocytes (Olig2+) and microglia (Iba1+)

**Fig. 11:**
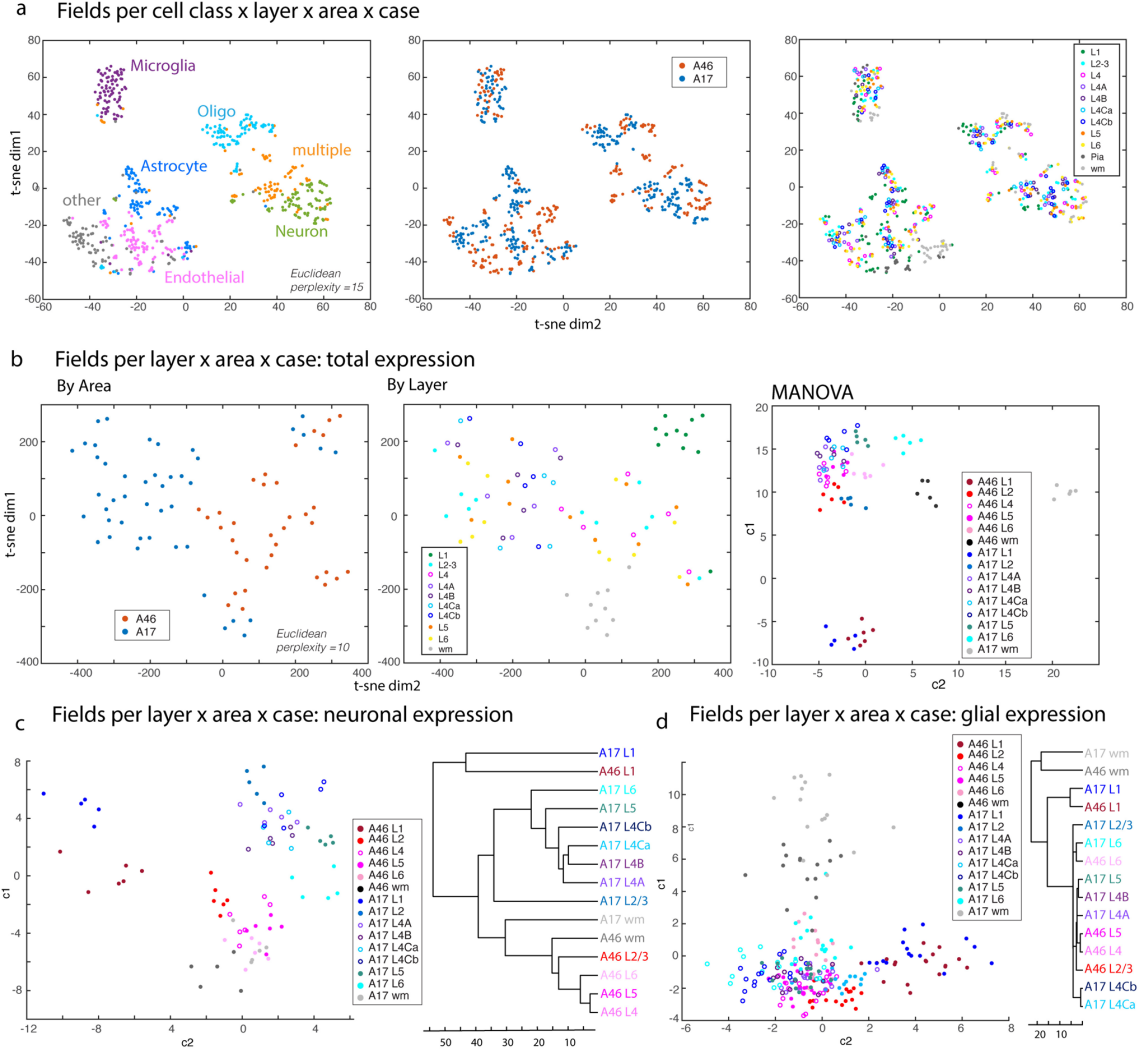
Cluster Analyses and MANOVA. **a** t-SNE plot of individual fields per cell type x layer x area x case; each field is an average of marker expression by cell type. t-SNE plot shows relative distances of fields (Euclidean distance, perplexity = 15) based on average expression Z intensity of 21 markers for each cell type, layer and area. **b** t-SNE plots (left and middle) and MANOVA (right) plot of first two canonical variables of individual fields per layer × area x case of averaged total marker expression (regardless of cell type). Plots are based on distance matrices from pair-wise correlations of 37 outcome measures, namely mean grayscale intensities (MGIs) of 21 markers and cell densities of 16 markers. **c** MANOVA (right) plot of first two canonical variables and dendrogram (left) of Euclidean distances between group means, of fields based on neuronal expression profiles and **d** glial expression profiles

All analyses were performed in RStudio 2022.12.0+353 (Posit teams 2022), except for clustering analyses performed in MATLAB. For each marker, we removed the top and bottom 0.2% of MGI values within each cell type; for microglia, which were present in lower numbers, we omitted the top and bottom 1% of MGI values. We used generalized linear mixed effects models (GLMMs) to explore interactions of our different variables of interest, using subject as a random effect blocking factor (Chang et al. 2022; Darian-Smith et al. 2013; Grafen and Hails 2002). We used one-way, two-way, and three-way ANOVAs as appropriate to determine whether there were differences in our variables of interest. Post-hoc analyses were conducted using Šidák-adjusted comparisons of estimated marginal means (emmeans). For all analyses, the significance level was α = 0.05, applying a Bonferroni correction for multiple comparisons. Two-way ANOVAs with independent factors of brain area and cortical layer were used for the dependent variables of density and average number of cell types, as well as MGI of individual neuronal markers. For the average number of cells of each marker-expressing subtype within a cell class, a three-way ANOVA was conducted to compare between-area, between-layer and between-subtype. For data analyses within layer 4, we performed separate one-way or two-way ANOVAs of dependent variables versus A46 and A17 sublayers. Statistical outcomes are presented in Supplementary Tables (4–12) associated with each of Figs. 3, 4, 5, 6, 7, 8, 9, 10, 11.

### Dimensional reduction, clustering and MANOVA analyses

To determine how distinct layers of each region are (dis)similar to each other based on neuronal and glial expression profiles of multiplex protein markers, we used dimensional reduction, clustering and MANOVA, as described (Gilman et al. 2017; Medalla et al. 2017; Fig. 11). First, we took the average normalized Z intensity of 21 markers across each cell segmented object classified either as Neuron, Microglia, Astrocytes, Oligodendrocytes, Endothelial, other, or multiple per field (layer × area × case). We then ran unsupervised clustering and dimensional reduction of individual fields per cell type to see if fields clustered by cell type. A distance matrix (based on Euclidean distances) was first computed based on the multidimensional dataset. Using this distance matrix, the multidimensional data was reduced to two dimensions via t-distributed stochastic neighbor embedding (t-SNE), which assigns a coordinate value to plot each data point in a two-dimensional space. In this dimensional reduction plot, the distances between each data point represent the similarities in the multidimensional properties—the closer the distance, the more similar the properties of the fields.

We then ran clustering, dimensional reduction and multivariate analysis of variance (MANOVA) analyses of fields per area × layer × case based on total combined features (Fig. 11b), and neuronal (Fig. 11c) vs glial (Fig. 11d) expression profiles of all the variables. For the total expression profile, we included 37 input variables based on normalized Z intensities (21 markers) and cell densities (16 markers) of markers of interest to cluster the distinct fields. Then, two separate sets of MANOVA were ran to classify fields: (1) based on a subset of neuron specific and pathological markers (15 intensity and 9 cell density measures); and (2) based on a subset of glial specific and pathological markers (13 intensity and 6 cell densities of glial specific and pathological markers; Suppl. Table 12). Each variable was normalized to z-score values, and a distance matrix (based on squared Euclidean distances) was computed.

We first ran t-SNE analyses on the full dataset and annotated the individual fields to visualize and see clustering based on categorical grouping variables as follows: by layer (1, 2–3, 4, 4A, 4B, 4Cα, 4Cβ, 5, 6), by areas (A46 or A17) by layer x area (A46: 1, 2–3, 4, 5, 6; A17: 1, 2–3, 4, 4A, 4B, 4Cα, 4Cβ, 5, 6). To validate the bottom-up t-SNE clustering scheme, a one-way MANOVA was used for supervised clustering, to compare the relative degree of influence each outcome measure contributes to the separation of the fields by area and layer, as described (Medalla et al. 2017). The properties were normalized to z-scores, and data points were plotted based on the first two canonical variables —weighted linear sums of all included variables— that best separate out the groups. For each canonical variable, the absolute value of the coefficient represents the degree of influence that each outcome measure contributes to the separation of the groups, with higher absolute values indicating a greater influence.

## Results

Multiplexed immunofluorescence (MxIF) was performed on 5-µm-thick paraffin embedded brain sections from 7 middle-aged monkeys (4 female, 3 male; Table 1). Subjects ranged from 14.5–20.3 years of age, and 4 of the monkeys contributed both A46 and A17 while 2 contributed only A46 and 1 contributed only A17 to this study (Table 1). A total of 28 different markers were assessed in these tissue sections (Table 2; Fig. 2). Photomicrographs of the 28 markers acquired on the same tissue section in 3 channels- Cy2/AF488, Cy3/AF555, and Cy5/AF647 at 20 × using the Cell DIVE™ MxIF platform are shown in Fig. 2a. For each tissue section, 400-µm wide regions of interest (ROIs) extending from the pial surface to 100 µm deep into the white matter (WM) from A46 and/or A17 were cropped from variably larger images (e.g., Fig. 2b). Laminar boundaries were delineated based on the layers established in the literature (Barbas and Pandya 1989; Rockland and Pandya 1979). For A17, layer (hereafter L) 4 was subdivided into 4 sublayers which were defined by (Lund et al. 1994; Suppl. Table 2). Laminar boundaries are demarcated by white lines in Fig. 2b.

### Cellular composition of the neuropil in A46 and A17

The density, overall proportions, number, and laminar proportions, of five major cell classes–neurons, endothelial cells, astrocytes, oligodendrocytes, and microglia—in A46 and in A17 are shown in Fig. 3 and associated statistical comparisons presented in Supplementary Table 3. As described in Methods, following segmentation of DAPI objects in QuPath, all objects were manually classified into one of these cell classes based on the presence of cell type-specific markers: HuD and/or NeuN for neurons; ALDH1L1 and/or GFAP for astrocytes; Iba1 for microglia; Olig2 for oligodendrocytes; collagen IV and vimentin for endothelial cells (which also were identified based on their association with vascular elements). A small percentage of DAPI objects in each region could not be unequivocally classified (4.72% in A46 and 4.42% in A17) and these objects were excluded from subsequent statistical analyses. A total of 30,139 cells were manually classified in six A46 ROIs and 36,858 cells were manually classified in five A17 sections.

The density of neurons in each cortical layer, the superficial WM and in each of the A17 L4 sublayers were calculated and compared (Fig. 3a, top left and middle). The mean density of neurons was significantly lower in A46 than in A17 for each of L2/3–6, including A46 L4 versus sublayers 4A, 4B, and 4Cβ of A17 L4 but did not differ between areas in L1 or in the WM. The mean density of neurons in L2/3–6 of A46 was typically approximately half that of A17. The mean density of neurons as a function of layer were similar within A46 while laminar differences were observed in A17. The mean density of neurons in L1 and WM of A17 were both significantly lower than in each of layers L2/3–6 and the mean density of neurons in L2/3 was significantly higher than that of L6.

The mean density of microglia was low in both A46 and A17 compared to other cell types (Fig. 3a, top right) and did not differ across areas or across layers in A46. However, in A17 the mean density of microglia was higher in both L1 and WM than in L2/3 and L5. The mean density of astrocytes (Fig. 3a, bottom left) did not differ between the two brain areas. The mean density of astrocytes in L1 was significantly higher than in L2/3 and L4 in A46 and significantly higher than L2/3, 4, 5 and 6 in A17. There were no differences in the mean density of astrocytes across the four sublayers of A17 L4. Oligodendrocyte (Fig. 3a, bottom middle) mean densities did not differ between areas. In both A46 and in A17, there were laminar differences in the mean density of oligodendrocytes, with a lower density in each cortical layer than in the WM. In the A46, L1 had lower mean densities of oligodendrocytes than did L6 while in A17 the mean density of these cells was lower in L2/3 than in L4. There were no differences in the mean density of oligodendrocytes across the four sublayers of A17 L4. Similarly, there were no differences across areas in endothelial cell mean density (Fig. 3a, bottom right). In the A46, endothelial cell mean density was similar across all layers but in A17 the mean density of endothelial cells was higher in L1 than in WM. There were no differences in the mean density of endothelial cells across the four sublayers of A17 L4.

The relative overall proportions based on the mean number of the five cell types in A46 and in A17 layers 1–6 are shown in Fig. 3b. The majority of cells were neurons in both areas, but the ratio of non-neuronal cells to neurons differed at 43:57 (0.75) in A46 and 27:73 (0.37) in A17. Specifically, non-neuronal cells comprised a higher proportion of all cells in A46 than they did in A17. The glia (astrocytes, oligodendrocytes, and microglia) to neuron ratio (GNR) was 32:57 (0.56) in A46 and 18:73 (0.25) in A17.

We then quantified the mean number of each of the five cell classes within each lamina and in the superficial WM to ascertain the relative distribution of different cell types as a function of layer (Fig. 3c). In both areas, the highest total number of cells were found in L2/3, specifically due to greater numbers of neurons compared to other layers. Within each area, the majority of the neurons were concentrated in L2–3, while L1 contained the lowest proportion of neurons in both areas, significantly lower than other layers.

We next assessed the relative laminar distribution of each cell type across the six cortical layers within each brain area (Fig. 3d). WM was excluded from these calculations because only 100 µm of WM could be sampled, rather than its entire vertical extent as with the cortical laminae. Between-area comparisons show that the mean proportion of neurons in L4 of A17 was much higher than that of A46 L4, while the proportion of neurons in L5 were higher in A46 than A17. When comparing A46 L4 and the sublayers of L4 A17, we observe a higher mean proportion of neurons in L4Cβ than in all other sublayers of A17 L4, as well as than in A46 L4. However, the mean proportion of neurons within A46 L4 was higher than that of A17 L4A and L4Cα.

Within both A46 and A17 each non-neuronal cell type had a distinctive laminar distribution. Oligodendrocytes were enriched in the deep layers of both regions and in L4 of A17 compared to other cell types, while astrocytes, microglia and endothelial cells exhibited a similar laminar distribution to neurons, except for a relatively greater proportion in L1 (Fig. 3d). For each cell type, we next examined the mean grayscale intensity (MGI) as a proxy for expression level and co-expression patterns of canonical cell type-specific markers across areas and layers (below).

### Distribution and marker intensity of HuD+ and NeuN+ neurons across layers in A46 and A17

We first examined coexpression patterns of HuD and NeuN, two canonical pan-neuronal markers. The mean proportions and marker intensity of HuD+ and NeuN+ neurons in A46 vs. A17 are shown in Fig. 4 and statistical comparisons provided in Suppl. Table 4. Representative photomicrographs across the pia-WM extent of A46 and A17 of a 17.3-year-old female monkey (Fig. 4a) show the varying intensities of HuD+ (green), NeuN+ (magenta) and double-labeled (white) neurons across layers. The relative overall proportions of neurons that were both HuD+/NeuN+ vs those that contained only HuD+ or only NeuN+ are shown in ring graphs (Fig. 4b). In both brain areas most neurons contained both HuD and NeuN (96% in A46; 91% in A17). The percentage of neurons that were NeuN+ only were slightly lower in A46 compared to A17 (3% vs 5%), while HuD + only neurons represented 4% in A17 compared to just 1% in A46. There was a distinctive pattern of HuD + /NeuN+, HuD+ only and NeuN+ only neurons across laminae and in WM in both brain areas as shown in laminae and WM relative proportions histograms (Fig. 4c, d). L2/3 contained the highest proportion of NeuN+ neurons in both brain areas followed by L1, with just a few NeuN+ only neurons being present in the deeper layers. By contrast HuD + only neurons were distributed more evenly across layers.

The MGIs of HuD and of NeuN in the entire population of HuD and/or NeuN labeled neurons are plotted in Fig. 4e. While both areas showed a near linear increase in HuD MGI from superficial to deep layers, the intensity of label in L2/3, L4 and L5 was significantly less in A17 compared to A46 (Fig. 4e, left). The NeuN MGI showed marked laminar specificity with levels dropping significantly in layers 4 and 5, most notably in A17 (Fig. 4e, right). This pattern can be readily seen in Fig. 4a (A17 middle panel). The relationships between HuD MGI and NeuN MGI in the total population of neurons from all subjects by cortical layer are shown in 2-D scatter plots (Suppl. Fig. 2). Except for L1 in A17 and WM in A46 there was a significant positive linear relationship between HuD and NeuN MGI in each layer. Within the layers that showed significant correlations for both areas (L2/3–6), A17 showed a stronger correlation of the two markers in all but L4 for which the opposite was true.

Additionally, we assessed the expression and intensity of MAP2 within HuD + and NeuN+ neurons (Suppl. Fig. 3; Suppl. Table 5). Zoomed-in representative photomicrographs of A17 of a 19.2-year-old male monkey (Suppl. Fig. 3a) show the presence of MAP2 (red) in the cytoplasm of HuD+ (green), NeuN+ (magenta) neurons. Most neurons across both brain areas were positive for MAP2 (Suppl. Fig. 3b–c). The MGI of MAP2 in HuD+ and in NeuN+ neurons was significantly lower in L4 than in the other layers in both brain areas (Suppl. Fig. 3d).

### Distribution and marker intensity of HuD+ GABAergic neurons containing GAD67, GAD65, PV and/or CB proteins across layers in A46 and A17

Within HuD+ neurons, we quantified the proportions and MGIs of pan-GABAergic neuronal markers, GAD65+ and GAD67+, as well as markers for specific inhibitory neuron subpopulations, parvalbumin (PV) and calbindin (CB) (Fig. 5; Suppl. Table 6). Representative photomicrographs in Fig. 5a show GAD65+ (yellow), GAD67+ (cyan), PV+ (green), and CB+ (magenta) neurons in a section of A17 from a 14.5-year-old male monkey. An important caveat related to these markers is that GAD67 and GAD65 do not label all inhibitory neurons and that GAD65 is primarily a synaptic not a somatic marker. Thus, the reported numbers are likely an underestimate of the total population of inhibitory neurons. The relative overall proportions of HuD+ neurons that were positive for either one or both GABA markers are shown in ring graphs (Fig. 5b). In both areas, the majority of HuD+ neurons were not GABAergic (i.e., they were negative for both GAD67 and GAD65). There were three distinct subpopulations of GABAergic neurons: GAD67+ only, GAD65+ only, and GAD65+/GAD67+ coexpressing neurons (8, 7, and 5% of the total neurons, respectively, in A46; and 5, 3, and 4% respectively in A17). Thus, GABAergic neurons comprised 20% of all neurons in A46 and 12% of all neurons in A17, consistent with previous estimates (Fig. 5b). The average excitatory (GAD-) to inhibitory (GAD+) neuron ratio (E:I) in the sampled area (L2/3-L6) was 4:1 in A46 and 7.5:1 in A17.

We then assessed GABAergic HuD+ neuron subpopulations based on GAD67/65 coexpression with PV and CB, two markers of functionally distinct inhibitory neuron subclasses (DeFelipe 1997; Hof et al. 1999; Ascoli et al. 2008). The majority of GAD67+ only, GAD65+ only, or GAD65+/GAD67+ expressing neurons did not express either PV or CB (Fig. 5c). Among those GABAergic neurons that did express CB or PV, most were GAD67+ only neurons and the remaining were GAD67+/GAD65+ (Fig. 5c). Additionally, we assessed the expression of calcium binding proteins CB and PV in HUD+/GAD− principal neurons within A46 and A17. Within both brain areas, the number of GAD− neurons negative for both PV and CB was significantly higher than those of GAD− neurons positive for either PV or CB only, or for both markers.

The relative number of GABAergic HuD+ neurons expressing PV and CB across laminae (L1 and WM were not included since there were no calcium-binding protein positive neurons observed) are shown in Fig. 5d. The three subpopulations of GABAergic neurons based on GAD65 and GAD67 expression showed distinct laminar distributions depending on area. Between-area differences were most pronounced for GAD65/GAD67 coexpressing neurons, with layer distributions showing opposite patterns. Interestingly, the number of GAD65+/GAD67+ neurons was highest in A17 L4Cβ compared to all other layers and sublayers. The laminar proportions of GAD67+ and GAD65+ neurons expressing CB and PV also differed by subtype. Specifically, for GAD67+ only neurons, the proportion expressing CB was greatest in the upper layers L2/3 for both areas. In contrast, the GAD67+/PV+ were more evenly distributed across the layers, with a greater proportion making up the total number of GAD67+ only cells in the middle to deep layers in both areas. The small population of GAD67/65 and CB coexpressing neurons were also preferentially located in L2/3. In contrast, the laminar distribution of GAD67/65 and PV coexpressing neurons was dependent on region, with enrichment in the upper layers of A46 but in the middle to deep layers of A17.

We then examined the MGIs of GAD67 (left) and GAD65 (right) within each cell for the total GAD+ (top), PV+ (middle) and CB+ (bottom) subpopulations of neurons across cortical layers (Fig. 5e). Within the total population of GABAergic neurons in both areas, the MGI of GAD67 was lower in L2/3 than in all other layers (Fig. 5e, top left). No differences in GAD67 intensity were found within the PV+ or CB+ subpopulations. The intensity of GAD65 differed between the two brain areas in L2/3 and L6, with A46 showing higher GAD65 MGI than A17 (Fig. 5e, top right). Within each brain area, GABAergic neurons showed a general pattern of higher GAD65 MGI in the upper to middle layers (L2-4) relative to the deep layers. Specifically, GABAergic neurons in A46 L2/3 and A46 L4 showed a higher MGI of GAD65 than those in A46 L5 and L6 (Fig. 5e, top right). In A17, GABAergic neurons in L6 showed lower GAD65 MGI than all other A17 layers. No significant differences in the intensity of GAD65 were observed in the PV+ subpopulation but in the CB+ subpopulation, a higher intensity of GAD65 was seen in A17 L4 than in A17 L6. When looking at the subpopulation of CB+ and PV+ GAD67+/GAD65+ neurons, we found that this positive correlation between GAD67 and GAD65 MGI was stronger in PV+ compared to CB+ neurons in both areas. We compared the MGI of PV+ versus CB+ within GABAergic HuD+ neurons in each area and layer and found that, similar to cell distribution, intensity per cell of CB+ vs PV+ had distinct laminar patterns. CB MGI showed greatest intensity per cell in L2/3 and decreased in L4. In contrast, PV MGI was relatively uniform across layers in A17, with a peak in L4 in A46 (Suppl. Fig. 4; Suppl. Table 5).

We assessed the relationship between the MGI of GAD67 and GAD65 in GAD+ neurons that were PV+ and CB+ using 2-D scatterplots (Suppl. Fig. 4, left). Both inhibitory neuron populations showed positive correlations in both brain areas. PV+ inhibitory neurons showed a stronger relationship between GAD65 and GAD67 in A46 (Suppl. Fig. 4, top left) while CB+ inhibitory neurons showed a stronger relationship in A17 (Suppl. Fig. 4, bottom left). Additionally, we assessed the MGI of PV and CB in GAD+ neurons positive for the respective marker in the different layers (Suppl. Fig. 4, right) and no laminar or areal differences were observed.

Finally, we examined the expression of the potassium channel Kv3.1 in HuD+ inhibitory neurons. Within all GAD67+ neurons, there were, on average, 43% of cells that were Kv3.1+ in A46 and 56% that were Kv3.1+ in A17. Kv3.1 channel expression was interneuron subtype specific; in A46 all GAD67+/PV+ cells were Kv3.1+ and in A17 95% of GAD67+/PV+ cells were Kv3.1+. In contrast, a lower proportion of GAD67+/CB+ cells were Kv3.1+, with 76% and 84% of these neurons being Kv3.1+ in A46 and A17, respectively (not shown).

### Distribution and marker intensity of astrocytes across layers in A46 and A17

Astrocytes were identified based on the expression of two canonical markers, GFAP and ALDH1L1 (Jurga et al. 2021; Khakh and Deneen 2019). Here we quantify the extent to which these markers colocalize and reveal the distribution of marker-specific astrocytes within A46 and A17 (Fig. 6; Suppl. Table 7). Representative photomicrographs showing ALDH1L1+ (green), GFAP+ (magenta) and double-labeled ALDH1L1+/GFAP+ (white) astrocytes in A46 and A17 from a 15.3-year-old male monkey are presented in Fig. 6a. The relative overall proportions of astrocytes that were GFAP+/ALDH1L1+ and those that stained only for ALDH1L1 or only for GFAP are provided in Fig. 6b. Most astrocytes contained both markers in both A46 and A17 (76% and 78% respectively). There was a slightly higher proportion of ALDH1L1+ only astrocytes in A46 compared to A17 (23% vs 18%). Very few astrocytes were GFAP+ only, with a higher proportion of these in A17 compared to A46 (4% vs 1% respectively). Bar graphs showing the relative numbers and proportions of GFAP+/ALDH1L1+, ALDH1L1+ only and GFAP+ only astrocytes across layers and in the white matter reveal distinct laminar patterns for the different populations of astrocytes (Fig. 6c, d). Astrocytes containing GFAP+ only were mostly limited to L1 and to the white matter. Of all astrocytes in L4 of A46, 52% were ALDH1L1+ only astrocytes; this type of astrocyte also comprised a significant proportion of L4B and L4Cα of A17 (48% and 40% respectively). Within A17 the highest proportion of ALDH1L1+ only astrocytes was found in L4Cβ, and the lowest proportion in L6 (45% vs 3%).

The MGI of GFAP and ALDH1L1 in GFAP+/ALDH1L1+ and of ALDH1L1 in ALDH1L1+ only astrocytes are shown in Fig. 6e. The patterns of intensity of these markers were similar in A46 and A17 with the only significant differences being a higher intensity of GFAP in GFAP+/ALDH1L1+ astrocytes in L2/3 of A17 compared to A46 and of ALDH1L1 in GFAP+/ALDH1L1+ astrocytes in L4 of A17 compared to A46. The MGI of ALDH1L1 in ALDH1L1+ only astrocytes in L5 of A17 was significantly higher compared to A46. In both brain areas the MGI of GFAP demonstrated a U-shaped laminar pattern with relatively high intensity in L1, low intensity in L2/3 and L4, then linearly increasing levels from L5 to L6, and highest intensity in the white matter. The pattern of ALDH1L1 MGI was the inverse of GFAP with, in both brain areas, a low intensity in L1 and WM and highest levels of intensity in L2/3 and 4. Within L4, ALDH1L1 intensity in GFAP+/ALDH1L1+ astrocytes in A46 differed from both L4Cα and L4Cβ of A17 while within A17, L4Cβ differed from both L4A and L4B. Additionally, ALDH1L1 intensity in ALDH1L1+ only astrocytes in A46 L4 differed from those in L4Cβ of A17 and within A17 the intensity of this marker differed from L4A vs both L4Cα and L4Cβ.

Linear regression analyses of ALDH1L1 and GFAP MGIs within astrocytes in each layer revealed a regional difference (Suppl. Fig. 5). Specifically, in L1 and L2/3, there was a positive correlation between ALDH1L1 and GFAP MGI in A17 but not in A46. Interestingly, in the middle deep layers (L4-L6), the positive correlation between these markers was weaker (A46 L6) or absent, or in the case of L5 A17, reversed. In L5 A17, astrocytes with greater ALDH1L1+ intensity exhibited lower GFAP+ intensity. In the white matter strong positive correlation between the markers was present in both areas.

Of note, the oligodendrocyte marker Olig2 was expressed in some astrocytes (Suppl. Fig. 6a; Suppl. Table 7). A large proportion of astrocytes in A17 (34%) but not A46 (4%) exhibited above-threshold values of Olig2 expression (Suppl. Fig. 6b). The Olig2+ astrocytes were mostly ALDH1L1+/GFAP+ or ALDH1L1+ only astrocytes with only a small number of GFAP+ only astrocytes in L1 and WM being Olig2+ (Suppl. Fig. 6b–d). The MGI of Olig2 in the different groups of Olig2+ astrocytes was also assessed and no significant differences between layers or areas were found.

### Distribution and marker intensity of oligodendrocytes across layers in A46 and A17

We then assessed coexpression of canonical markers for oligodendrocytes, and their subpopulations and state of differentiation (Fard et al. 2017; Kuhn et al. 2019). The proportions and marker intensity of Olig2+, CNPase+ and BCAS1+ oligodendrocytes in A46 compared to A17 are shown in Fig. 7 and associated statistical comparisons presented in Suppl. Table 8. Photomicrographs showing Olig2+ (green), CNPase+ (magenta) and BCAS1+ oligodendrocytes in A46 and A17 (long panels) from a 15.3-year-old male monkey (Fig. 7a). Olig2 was present in all oligodendrocytes as a nuclear marker. CNPase and BCAS1 by contrast are proteins associated with myelinating oligodendrocytes, which are localized within oligodendrocyte processes and cytoplasm (Fig. 7a). BCAS1 in particular is thought to mark newly myelinating subpopulation of oligodendrocytes (Fard et al. 2017; Kaji et al. 2020). The labeling thus appears fibrillar in the neuropil, due to the presence of these proteins where oligodendrocyte processes associate with myelin and can also be clearly seen in the perinuclear cytoplasm and processes of Olig2+ oligodendrocytes (Fig. 7a).

The relative overall proportions of oligodendrocytes that were Olig2+ only vs Olig2+/CNPase+ only, Olig2+/BCAS1+ only or contained all 3 oligodendrocyte markers are shown in Fig. 7b. The proportion of oligodendrocytes that contained just the Olig2 marker was similar across A46 and A17 (78% vs 69% respectively). Some Olig2+ oligodendrocytes also stained for CNPase in both brain areas, comprising 19% of the oligodendrocytes in A46 and 25% in A17. BCAS1+ oligodendrocytes were relatively rare in both areas but were observed at a higher proportion of the total population of oligodendrocytes in A17 (4%) compared to A46 (2%). Oligodendrocytes containing all 3 markers were observed in very small quantities in both A46 and A17, comprising 1% and 2% of the total population, respectively. Histograms showing the relative proportions and average counts of oligodendrocytes staining for the 3 markers across layers are shown in Fig. 7c, d. WM was not included in estimates since the CNPase+ and BCAS+ myelinated fibers were too dense to allow unequivocal assessment of these markers’ colocalization with Olig2. Layer 1 oligodendrocytes stained exclusively with Olig2, and this group also represented most of the oligodendrocytes in L2/3 (Fig. 7c). Olig2+ only oligodendrocytes also were predominant in L4 of A46 and L4A and L4B of A17. Layer 4C in A17 marked the appearance of a large proportion of Olig2+/CNPase+ only cells with 20% of all Olig2+/CNPase+ only cells in L4Cα and 43% in L4Cβ. The proportion of Olig2+/CNPase+ oligodendrocytes did not differ in L5 or L6 in either brain area.

The MGIs of Olig2 in all Olig2+ oligodendrocytes, CNPase in all Olig2+/CNPase+ oligodendrocytes and of BCAS1 in all Olig2+/BCAS1+ oligodendrocytes are shown in Fig. 7e. The intensity of Olig2 was markedly greater in A17 compared to A46 oligodendrocytes in every layer except L1. The intensity of Olig2 did not differ across layers in either brain area. CNPase intensity in CNPase+ oligodendrocytes was lower in A46 than in A17 L4 and L5, but there was no significant difference in the intensity of this marker in any other layers across regions. Finally, BCAS1 staining intensity in BCAS+ oligodendrocytes did not differ between or within areas.

Linear regression reveals distinct relationships of Olig2 expression per cell with BCAS1 and CNPase intensity (Suppl. Fig. 7). The strongest correlations between MGIs of oligodendrocyte markers were found in L1, wherein BCAS1 intensity exhibited a positive correlation with Olig2 intensity in A17 (Suppl. Fig. 7a); but CNPase was significantly negatively correlated with Olig2 intensity only in L1 of A46 (Suppl. Fig. 7b). In addition, there was a significant positive correlation between BCAS1 and Olig2 MGI in L2/3–6 of A46, and in L4 and L6 of A17 (Suppl. Fig. 7a).

### Fibrillar components of the neuropil in A46 and A17

Photomicrographs showing 6 fibrillar markers—BCAS1, CNPase, MAP2, MBP, pan-pNF and npNF- in A46 and A17 (long panels) from a 17.3-year-old female monkey are shown in Fig. 8a. Line graphs showing (from top to bottom) the normalized and z-scored intensity of BCAS1, CNPase, MBP, MAP2, pan-pNF, and npNF as a function of normalized depth in the two brain areas reveal patterns of myelinated axons, axons, and dendrites in the neuropil (Fig. 8a). The MGIs of BCAS1, CNPase and MBP across cortical layers, demonstrate that the laminar expression pattern of oligodendrocyte markers, BCAS1 and CNPase, mirrored that of MBP; the three markers showed concomitant increasing intensities with increasing depth/distance from pia in both areas (Fig. 8b, top 3 panels; Suppl. Table 9). In addition, consistent with classic myeloarchitectural observations, A17, but not A46, displayed a prominent a non-linear peak of these myelin-oligodendrocyte markers in L4.

Within-area comparisons showed that BCAS1 MGI in A46 L1 differed significantly from all other layers, while in A17 L1 differed from L4, L6 and WM and L2/3 differed from WM. CNPase intensity of A46 L1 was lower than all other layers, that of L2/3 differed significantly from all other layers, having a higher MGI than that of L1 but lower than L4-WM, while A46 WM was significantly higher than that of every layer except L6. Within A17, L1 and L2/3 intensity of CNPase and MBP was significantly lower than that from L4, L5, L6 and WM while that of the WM was higher than all other laminae for CNPase but not in L6 for MBP. MGI of MBP in L1 of A46 was lower than all other A46 layers while L2/3 was lower than that of L5, L6 and WM and intensity in the WM was also higher than that of L4. No differences between areas were observed for BCAS1, CNPase, MBP. We then examined the laminar distribution of neurofilament proteins that primarily label axons (pan-pNF) and dendrites (npNF and MAP2) in the neuropil. The axonal marker pan-pNF showed a laminar expression pattern comparable to myelin/oligodendrocyte markers (Fig. 8b). In both areas, the MGI of pan-pNF was generally lower in upper layers compared deep layers and the WM. The MGI of the non-phosphorylated neurofilament protein, npNF, which is enriched in proximal dendrites of a subset of L3 and L5 neurons, only showed significant laminar differences within A46, with L1 having lower intensity than L4, L5, L6 and WM. The microtubule associated protein marker MAP2 showed similar MGIs in L1 and L2/3 of the two brain areas and followed the same trend of decline in L4 of both A46 and A17 before increasing in L5 and L6 (Fig. 8b, bottom left panel). Between-area analyses showed no significant differences in pan-pNF, npNF and MAP2 MGIs.

### Characteristics of vascular profiles in A46 and A17

Three different vascular markers—collagen IV, vimentin and SMA—were used to identify blood vessels in A46 and A17 (Fig. 9a, left, long panels) and at a higher zoom from the A46 (Fig. 9a, right, square panels) from a 17.6-year-old female monkey. Vessels with a perimeter larger than 400 µm were considered outliers and excluded from analysis. Statistical comparisons are presented in Suppl. Table 10. Circularity was assessed as a control to ensure that plane of section did not differ significantly in the two brain areas prior to further characterization. Figure 9b (left) is a histogram showing that the distribution of all vessels (as defined by collagen IV staining) in A46 vs A17 was very similar; indeed, the mean circularity of vessels did not differ across layers in either brain region and did not differ between regions (Fig. 9b, middle panel). The mean circularity of vessels also did not differ between A46 L4 and between any of the sublayers of A17 L4 (Fig. 9b, right panel). Having established that vessels were not differentially sectioned with regard to their long axes in the two regions, we next assessed the perimeter of the vessels as a proxy for their size to compare the two brain areas and to assess potential differences in the size of vessels between layers. The distribution of vessel perimeters did not differ significantly between brain areas and most vessels (53%) had a perimeter of less than 50 µm with a range of 11.05–386.61 µm (Fig. 9c, left panel). Furthermore, the mean perimeter of vessels increased linearly from L1 to the WM and this pattern was seen in both A46 and in A17 (Fig. 9c, middle panel). The mean perimeter of vessels did not differ between A46 L4 and between any of the sublayers of A17 L4 (Fig. 9c, right panel).

To gain insight into the area of neuropil occupied by vessels in the two brain areas we assessed the area occupied by vessels across layers (Fig. 9d, left two panels) as well as the fraction of each layer occupied by vessels (Fig. 9d, right two panels). Based on area occupied, A46 L1 and WM were significantly less vascularized than A46 L2/3 and L6 while the same was true for A17 L1 and WM when compared to A17 L2/3 and L4. Vascular occupancy of the neuropil did not differ significantly between brain areas except in L4 which was significantly more vascularized in A17 than in A46. Within area comparisons showed that vascular occupancy of the neuropil in L4 was greater than in L1, L5, and WM in A17, while in A46 L4 was less occupied with vessels than L2/3 and 6. Interestingly, there was also a marked difference in the area occupied by vessels within the sublayers of L4 in A17 with L4Cβ showing a higher area occupied but not fraction occupied, reflecting the larger extent of this sublayer. The fraction of each layer and WM occupied by pixels positive for collagen IV (left), vimentin (middle) and SMA (right) shown in Fig. 9e demonstrate that the only difference in the laminar distribution of these marker-specific vessels was found within A17, where L1 has a greater average positive fraction of pixels than WM.

### Presence of pathology markers within neurons, astrocytes, oligodendrocytes, and microglia

We then assessed the co-expression of cell type-specific proteins with seven different markers of oxidative stress or misfolded proteins—Aβ, pTau, Cleaved Caspase3, S6, TSPO, 3NT and TDP43 (Fig. 10). These markers were prevalent in all neurons, and more specifically in excitatory (negative for GAD67) neurons (Fig. 10b, top left and middle; Suppl. Table 11). In excitatory neurons, 5 of the 7 markers were present at significantly higher intensity in of A46 than of A17, with only Cleaved Caspase3 being present at the same intensity and TSPO being present at significantly higher intensity in A17 neurons. For the inhibitory neuron group (positive for GAD67), intensities were higher in A46 than in A17 for 4 of the markers (pTau, Cleaved Caspase3, 3NT, and TDP43) while no differences between areas were observed for the other marker intensities (Fig. 10b, top right; Suppl. Table 11). Differences in mean intensity of pathological markers in astrocytes only differed between areas for Aβ, TDP43, and TSPO with the former two being higher in A46 and TSPO being higher in A17 (Fig. 10b, bottom left; Suppl. Table 11). In oligodendrocytes the mean intensity of Aβ, S6 and TDP43 was lower in A17 than in A46 while that of pTau and 3NT was higher in A17 than in A46, with the remaining markers showing no differences between areas A17 (Fig. 10b, bottom middle; Suppl. Table 11). The mean intensity of pathological markers in microglia did not differ between brain areas (Fig. 10b, bottom right; Suppl. Table 11).

### Dimensional reduction and clustering reveal cortical regional and laminar organization based on multivariate neuronal and glial proteomic profiles.

Using dimensional reduction, clustering and MANOVA, we assessed how distinct layers of each region are (dis)similar to each other based on multiple neuronal and glial expression profiles. First, we took the MGI of 21 markers for each cell type layer and area. We then ran unsupervised clustering and t-SNE dimensional reduction and found that the fields clustered most robustly according to cell type (Fig. 11a). There is also some subclustering of fields by area, within cell type profiles.

We then ran separate clustering and MANOVA analyses of fields per area x layer x case based on total (Fig. 11b), and neuronal (Fig. 11c) vs glial (Fig. 11d) expression profiles of all the variables. We included 35 input variables based on normalized Z intensities (21 markers) and cell densities (14 markers) of markers of interest (Suppl. Table 12). MANOVA based on total expression of both neuronal and glial profiles significantly segregated, fields per area x layer x case into six groups, based on five significant canonical variables (c1, c2, c3, c4, c5; p < 0.05, Suppl. Table 12). Specifically, when a combination of total neuronal and glial features was considered together, the first canonical variable (c1) separated A17 WM from the rest of the groups. The second canonical variable (c2) separated L1 fields from the rest of groups; followed by a between-area separation of A17 L1 from A46 L1 based on the 3rd canonical variable. The 4th and 5th canonical variables then segregated A17 L2/3 and A46 WM, respectively, from the rest of the fields. These indicates that based on the combined expression of neuronal and glial markers, A17 WM and fields in L1, followed by A17 L2/3 and A46 WM, were the most dissimilar than the rest of the cortical fields examined. Interestingly, the clustering of fields × area × layer seems to be more driven by neuronal than glial features. Among all variables, the density of neurons, specifically HUD+, pyramidal/stellate neurons and MAP2+ neuron densities, had the highest value of coefficients that most influenced the significant canonical variables (c1–c5) that determined the separation of clusters. However, among glial markers, the density and intensities of CNPase oligodendrocyte marker and GFAP astrocyte markers are significant drivers of clustering, especially in the distinction of L1 from the rest of the fields (c2, see Suppl. Table 12).

We then assessed the independent effects of neuronal expression and glial expression profiles on the similarity of layers and areas by running two distinct MANOVAs with two sets of neuronal versus glial features. To assess clustering of fields based on neuronal expression, the set of outcome measures for MANOVA included 15 intensity and 8 cell densities of neuron specific and pathological markers (Suppl. Table 12). Based on this neuronal expression profile, we found a strong clustering of the fields by area and layer, with significant separation of 4 clusters based on 3 significant canonical variables (Fig. 11c, MANOVA p < 0.05; Suppl. Table 12). The first segregation based on neuron features was between L1 from both areas (c1) and the rest of the fields (Fig. 11c, Suppl. Table 12). Then inter-areal subclusters emerge, with the 2nd canonical variable (c2) segregating A46 L1 from A17 L1, and the 3rd canonical variable (c3) segregating the rest of the A17 layers from A46 layers. The clustering also showed that between-layer dissimilarities were more pronounced in A17 than A46, with distances between A17 layers greater than A46 layers. Among all neuronal features, cell densities of HuD+, pyramidal/stellate and MAP2+ neurons had the strongest influence on separations based on the first 3 significant canonical variables (Suppl. Table 12). Canonical variables were also driven by GAD65+ intensities and density of npNF+ neurons.

To assess clustering of fields based on glial expression, MANOVA included 13 intensity and 6 cell densities of glial specific and pathological markers. Similar to neuronal features, glial expression profiles only had three significant canonical variables that segregated four distinct clusters (Fig. 11d, MANOVA p < 0.05; Suppl. Table 12). However, glial expression profiles did not systematically discriminate between areas and between all layers compared to neuronal expression profiles. The two main clusters were between gray and white matter (separated by c1). Among all glial outcome features, the intensities of ALDH1L1 and S6 as well as the densities of oligo markers Olig2 and BCAS1, were the strongest drivers of this WM/GM segregation. Then within the grey matter cluster, L1 from both areas separated into a distinct cluster from other fields (c2), which was driven mainly by differences in glial cell densities of astrocytes and oligodendrocytes. Then a significant segregation based the third canonical variable with A17 L2/3 and the rest of the fields (c3). Interestingly, this last separation was mainly driven by intensity of the pathological markers pTau and Cleaved Caspase3 within glial cells (Suppl. Table 12).

In summary, the neuronal and glial features segregated the groups distinctly, with between-area clustering more pronounced based on neuronal profiles than on glial profiles. Not only did neuronal features significantly cluster between distinct groups based on area × layer, but the differences (Euclidean distances) between the clusters were greater (Fig. 11c, d). These data show that neuronal features are more divergent across areas and layers compared to glial features.

## Discussion

We used iterative MxIF to compare the distribution of 28 cellular and subcellular protein markers across laminae in the gray matter neuropil and superficial white matter of the primary visual cortex (A17) and the dorsolateral prefrontal cortex (A46) of the adult rhesus monkey. Recent single cell transcriptomic studies (including sNucSeq) have yielded data that are predictive of phenotypic variation and point to significantly greater cellular diversity in the mammalian neocortex than previously appreciated (Tasic et al. 2018; Bakken et al. 2021; Berg et al. 2021; Khrameeva et al. 2020; Krienen et al. 2020; Lei et al. 2022; Lein et al. 2017; Yao et al. 2021; Zhu et al. 2018; Jorstad et al. 2023). As many as 24 transcriptomically distinct subclasses of neuronal and non-neuronal cells in diverse neocortical regions were recently described with differences between areas primarily due to differences in the distribution of diverse subclasses of excitatory neurons (Jorstad et al. 2023). In the present study we similarly found that differences between layers within areas and between areas were primarily accounted for by differences in neuronal number and distribution.

sNucSeq methods are based on the presence of RNA molecules in the nucleus, and thus only indirectly point to the potential presence or levels of translated proteins in various cellular compartments. Since translation, protein turnover and posttranslational modifications often differ between cells, transcript levels and cognate protein levels do not necessarily correlate (Nie et al. 2007; Liu et al. 2016). Highly polarized cells such as neurons add additional complexity to understanding the relationship between nuclear RNA expression and cellular phenotype (Moritz et al. 2019) since many proteins are specifically localized to cytoplasmic regions in the cell body, dendrites, and axonal processes. Further, whether transcriptomic features are indicative of functionally distinct cell types or perhaps of different states of the same cell is an important unanswered question.

While gene expression studies are highly informative, the compilation of a comprehensive brain cell census requires -in addition to morphological and functional characterization of individual cells across the lifespan- the direct assessment of the expression of diverse proteins per se. In the present study, we assessed 28 cellular and subcellular protein markers. Fourteen of these markers were specific for neurons and non-neuronal cells, and seven identified markers of oxidative stress or misfolded proteins. A further four markers labeled myelinated fibers and neurofilament proteins in axons or dendrites while three markers labeled vascular components of the neuropil and white matter.

A rich dataset of 66,997 precisely localized cells were segmented as DAPI objects and manually classified—30,139 cells in A46 from 6 subjects and 36,858 cells in A17 from 5 subjects. MxIF on the same tissue section(s) has the benefit of revealing protein expression intensities in diverse morphological compartments beyond the nucleus and they are capable of much greater spatial resolution and capture efficiency since transcripts that are present at low levels cannot be detected with sNucSeq (Thrupp et al. 2020; Cardona-Alberich et al. 2021).

### Density, distribution and proportions of neurons and non-neuronal cells

The density of neurons identified with HuD and/or NeuN in L2-6 was approximately twice as high in A17 compared to in A46, while the density of neurons in L1 and in WM (very low in both areas) did not differ. This interareal difference in neuronal density is consistent with previous stereological estimates (e.g. Hilgetag et al. 2016). In particular, the density of neurons in the expanded L4 was about three times greater in A17 than in A46 with a lower density of neurons in L5 of A17 compared to A46. In both brain regions neurons were the principal cell type present in the gray matter neuropil, but the ratio of non-neuronal cells (glial cells and endothelial cells) to neurons differed significantly at 0.75 (43:57) in A46, compared to 0.37 (27:73) in A17. In both brain areas endothelial cells comprised ~ 30% of the non-neuronal cell population and did not exhibit significant areal or laminar differences, consistent with previous estimates in other brain areas and species (review, von Bartheld et al. 2016). The GNR was 32:57 (0.56) in A46 and 18:73 (0.25) in A17. The GNR for A46 is somewhat lower than the value of ~ 0.9 reported previously for prefrontal area 46 in the rhesus monkey (Dombrowski et al. 2001). Similarly, the GNR in A17 was lower than the previous report of ~ 0.49 in the primary visual cortex in the rhesus monkey (O’Kusky and Colonnier 1982).

The higher glia to neuron ratios observed in previous studies compared to this study is likely due to methodological differences. Earlier studies primarily employed Nissl staining in which it is notoriously difficult to discriminate small neurons from glia; thus, it is possible that the number of neurons were underestimated, and the number of glia overestimated with these methods. By using immunofluorescence for both NeuN and HuD to identify neurons and multiple markers specific for glial cells we circumvent these issues by visually identifying cells with highly specific cell markers.

This is the first study to systematically compare the proportions of different glial cells across A17 and A46 using immunofluorescence for specific glial markers. Oligodendrocytes comprised 46.9% and 50% of all glial cells in A46 and in A17 respectively, with astrocytes comprising 43.8% and 44.5% of all glial cells in A46 and in A17 respectively. Microglial cells identified using Iba1 comprised 9.3% of glial cells in PFC and 5.5% in A17. Our findings are only partially consistent with earlier studies in that we observed a higher proportion of oligodendrocytes and a lower proportion of astrocytes. For example an early study by O’Kusky and Colonnier (1982) quantified the following proportions of glial cells in macaque V1: 65% astrocytes, 29% oligodendrocytes, and 6% microglia and a subsequent electron microscopy study reported similar proportions of 57% astrocytes, 35% oligodendrocytes, and 8% microglia within A17 of the rhesus monkey (Peters et al. 1991). What accounts for the 10–15% difference in glia proportions between studies? Again, this could be due to the technical difficulty in discriminating glial subtypes with Nissl (O’Kusky and Collonier 1982) and the small sample area available for electron microscopy analyses (Peters et al. 1991).

### Distribution and intensities of HuD- and NeuN-positive neurons

Neurons were identified as DAPI objects containing the RNA binding protein HuD, the nuclear protein NeuN or most commonly, containing both markers. In both A46 and A17 most neurons (96% and 91% respectively) contained both HuD and NeuN but at markedly different intensities. HuD-only neurons were scarce in both A46 (1%) and A17 (4%) and NeuN-only cells were similarly sparse, comprising just 3% and 5% of the total neuronal population in A46 and A17 respectively. While HuD-only neurons were relatively uniformly distributed across laminae in both areas, NeuN only neurons were confined primarily to supragranular layers 1–3 in both regions.

The signal intensity of HuD and NeuN differed significantly in the two regions. HuD signal increased linearly from L1 to L6 in both brain regions and for L2–5 was significantly higher in A46 compared to A17. HuD plays an important role in neurogenesis, neuronal morphology and neuronal signaling by regulating the metabolism of target mRNAs through modifications of stability, translation, splicing, and adenylation (Jung and Lee 2021). NeuN (also known as Fox 3), a nuclear protein that is specific to post-mitotic neurons, acts as a regulator of neuronal differentiation (Mullen et al. 1992; Kim et al. 2009; for review, see Gusel’nikova and Korzhevskiy 2015). Thus, the different expression levels of these neuronal markers in different layers and across regions is noteworthy and has significant implications for potential downstream protein expression and functional differences.

NeuN has been broadly used both to discriminate neurons from non-neuronal cells histologically (Gittins and Harrison 2004; Halene et al. 2016; García-Cabezas et al. 2016) and for cell-sorting in transcriptomic studies (Martin et al. 2017; e.g., Jorstad et al. 2023). Data presented here and in previous studies clearly indicate that NeuN labeling can be sporadic, e.g., only 18–57% of neurons were NeuN-positive in histological sections from human cortex (Lyck et al. 2009), and thus sound a cautionary note about relying solely on NeuN for identification or sorting of neurons. The functional relevance of the differences in intensity of staining for the two neuronal markers is currently unclear and awaits further investigation of the relevance of these modifiers of RNA translation in specific cell types. Given that the distribution patterns of these two markers can evidently be quite different, further evaluation may uncover differentiated roles in protein expression. In A17, this might reveal a highly layer-specific pattern.

### Ratio of inhibitory to excitatory neurons and other features of interneurons

Interneurons were identified based on the presence within HuD+ neurons of the ubiquitous marker for GABAergic neurons GAD67 (responsible for GABA synthesis unrelated to neurotransmission) and GAD65, (responsible for GABA synthesis for neurotransmission, so primarily present at nerve terminals). Importantly GAD67 and GAD65 do not label all inhibitory neurons thus the reported numbers are likely an underestimate of the total population of inhibitory neurons. HuD+ neurons that were positive for GAD67, GAD65 or both were relatively similar in distribution between the two areas: 8, 7, and 5% of the interneurons in A46 and 5, 3, and 4% of the interneurons in A17 (both areas: L1–6).

A recent report showed highly distinctive excitatory to inhibitory neuron ratios in V1 compared to PFC in human with ratios of 4.5:1 vs 2:1 respectively (Jorstad et al. 2023). We similarly observed a higher E:I ratio in A17 compared to A46 at 7.3:1 and 4:1 respectively. Hornung and De Tribolet (1994) also reported a ratio of 4:1 in human frontal cortex. The interneuron specific markers PV and CB overlapped with GAD67 and GAD67+ GAD65 markers but not with cells that were GAD65+ only and which were localized primarily to L1. In this study we found that only about a third of GAD67+ neurons labeled with PV or with CB, indicating that a substantial number of interneurons are likely of the calretinin (CR, one of the markers for which staining failed in this study), nitric oxide, or undetermined subtypes.

### Distribution and intensities of glial cell markers

We assessed the density, distribution, and marker intensity of each major glial class. Both brain areas showed a similar distribution and intensity of Iba1 staining of sparse microglia, with the highest density in L1 and in WM, and consistently low density in L2–6 (peaking in L5).

*Astrocytes.* Many astrocytes were positive for both ALDH1L1 and GFAP, with this group comprising 76% and 78% of the astrocyte population in A46 and A17 respectively. Nearly all other astrocytes were ALDH1L1+ only. ALDH1L1+/GFAP+ astrocytes were relatively uniformly distributed across cortical layers in both brain regions except for a notable decrease in density of this marker-specific class in A17 L4, particularly in L4A and B, consistent with a recent observation in human V1 (Jorstad et al. 2023). While ALDH1L1+ only positive astrocytes were quite uniformly distributed across layers in A46, this population was heavily localized to L4 (and specifically L4Cβ) in A17.

GFAP is routinely employed as a marker for astrocytes, but a relatively small proportion of astrocytes (1% in A46, 4% in A17) expressed only GFAP. These cells were localized almost exclusively in L1 and the superficial white matter in both brain areas. This study and others highlight the importance of employing multiple markers for a more thorough assessment of the diverse astrocyte populations, which are typically divided into protoplasmic astrocytes seen throughout the gray matter, interlaminar restricted to L1 and fibrillar, observed primarily in the white matter (Falcone et al. 2019, 2021, 2022; review, Escartin et al. 2021; Jurga et al. 2021). The intensity of the markers within the total population of astrocytes (containing either marker) exhibited inverse intensity distributions, being U-shaped for GFAP and bell-shaped for ALDH1L1, suggesting a more prominent role for GFAP in L1 (interlaminar astrocytes) and deep layers (fibrous astrocytes) and more important role for ALDH1L1 in middle layers (protoplasmic astrocytes).

We observed that a significant proportion of astrocytes in A17 (34%) but not A46 (4%) were also weakly positive for the oligodendrocyte marker Olig2. The presence of Olig2 in astrocytes has been previously reported in the adult mouse CNS (Wang et al. 2021)*.* The presence of robustly Olig2+ astrocytes as a substantial subset of astrocytes is consistent with the idea of further functional specialization and potential subclassification of this type of glial cell and highlights the utility of multiple marker assessment in cell census studies. As has been previously suggested (Wang et al. 2021), astrocytes and oligodendrocytes may share common properties and the interplay of Olig2 in regulating both glial cell types offer intriguing avenues for further investigation.

*Oligodendrocytes*. Approximately half of the glial cell population in both areas was composed of Olig2+ oligodendrocytes (that did not express astrocytic markers or Iba1). These were present in approximately equivalent distributions across layers in A46, and were most dense in L4 of A17, as expected for this heavily myelinated layer. Olig2 staining intensity was significantly more intense in A17 than in A46 in all layers except L1.

The subclass of CNPase-positive oligodendrocytes was heavily prevalent in L4 of A17 (comprising half of the population of oligodendrocytes in L4C) but was sparse in L4 of A46. CNPase is associated with 4% of the total myelin protein in the brain and has been established to be primarily expressed in actively pre-myelinating and myelinating oligodendrocytes (Verrier et al. 2013). CNPase acts to metabolize the mitochondrial toxin 2′,3′-cAMP to 2-AMP in glial cells (Myllykoski et al. 2012). By reducing this toxin and increasing levels of the neuroprotectant adenosine, CNPase+ oligodendrocytes may protect white matter—particularly in highly metabolically active V1—from neurodegenerative processes.

A relatively low proportion of Olig2+ oligodendrocytes were also positive for the marker BCAS1 (2% and 4% in A46 and A17, respectively) and similarly for all 3 oligodendrocyte markers (1% and 2% in A46 and A17, respectively). The small population of BCAS1+ cells was localized throughout the layers except L1. BCAS1 (breast-carcinoma amplified sequence 1) is a specific marker for oligodendrocytes that are typically only transiently present during oligodendrogliogenesis and active myelination/remyelination (Fard et al. 2017; Pol et al. 2017). Since the brain tissue studied was from healthy adult macaques, it is to be expected that the expression of immature BCAS1+ oligodendrocytes was low.

### Markers of oxidative stress and misfolded proteins

As noted, tissue was obtained from healthy adult monkeys with no known pathology or neurodegenerative disease. However, it is known that a broad variety of markers that are upregulated in disease are present, albeit at significantly lower levels, in a baseline state in a variety of cells. Here we assessed levels of 5 markers of oxidative stress or free radicals (Cleaved Caspase3, S6, TSPO, 3NT and TDP43) as well as two markers of misfolded proteins (Aβ and pTau) across neurons, astrocytes, oligodendrocytes, and microglia. The mean normalized intensity of the markers in glial cells did not differ between regions. However, the intensities of 5 of the 7 markers (all markers except Cleaved Caspase3 and TSPO) were higher in A46 than in A17 neurons. The higher levels of markers often associated with pathological states in A46 compared to A17 may be associated with the selective vulnerability of association cortical areas compared to primary sensory regions during normal aging and in neurodegenerative diseases (Braak and Braak 1991; Grothe et al. 2018; Hof and Morrison 1990; Hof et al. 1990; Mrdjen et al. 2019). Higher baseline levels of oxidative stress and misfolded proteins could in part be responsible for selective vulnerability of frontal compared to occipital regions.

## Conclusions and future directions

Taken together, data from this study demonstrate the power of the MxIF approach to reveal nuanced and granular characteristics of proteins expressed in the brain and their potential interactions. Our multivariate clustering and dimensional reduction analyses based on large scale multiplexed protein expression data revealed that neuronal proteomic expression profiles are strong determinants of regional and laminar differences. In contrast, glial protein expression profiles influenced primarily laminar differences within each area. Neuronal proteomic features significantly clustered groups by area and layer, and the differences between the clusters were greater compared to clustering based on glial protein expression. These data show that neuronal features are more divergent across areas and layers compared to glial features, consistent with recent transcriptomic studies (Jorstad et al. 2023; Chiou et al. 2023).

Notably, protein-focused studies such as this, together with genetic and transcriptomic approaches, offer promising approaches for linking cellular and other (fibrillar, vascular, intracellular protein) features with function. It should be kept in mind that single cell transcriptomic and proteomic data, while important for cell classification schemes, do not capture the full complexity of cellular diversity nor spatial relationships in the microenvironment. Unique morphological and physiological properties, and developmental history are also key phenotypic characteristics to consider. Proteomic or transcriptomic data may be associated with, but do not reveal, major morphological differences, such as soma size of neurons among cortical regions, marked differences in spine number/density, axonal arborization patterns and connections, or different excitability properties (Elston 2000; Elston et al. 2001; Elston and DeFelipe 2002; Hsu et al. 2017; Medalla and Luebke 2015; Gilman et al. 2017; Luebke 2017; Medalla et al. 2017). We nevertheless are optimistic that further data along these lines will advance our understanding of the complex underpinnings of the diverse functional capacities of cortical regions.

## Supplementary Information

Below is the link to the electronic supplementary material.

Supplementary file1 (PDF 9701 KB)

Supplementary file2 (PDF 61 KB)

Supplementary file3 (PDF 35 KB)

Supplementary file4 (PDF 24 KB)

Supplementary file5 (XLSX 412 KB)

Supplementary file6 (XLSX 524 KB)

Supplementary file7 (XLSX 505 KB)

Supplementary file8 (XLSX 353 KB)

Supplementary file9 (XLSX 62 KB)

Supplementary file10 (XLSX 360 KB)

Supplementary file11 (XLSX 146 KB)

Supplementary file12 (XLSX 19 KB)

Supplementary file13 (XLSX 13 KB)

## Data Availability

Enquiries about data availability should be directed to the authors.
