# Supplementary material for: Proteomic features of gray matter layers and superficial white matter of the rhesus monkey neocortex: comparison of prefrontal area 46 and occipital area 17
Source: Brain Struct Funct. 2024 Jun 28;229(7):1495–525. doi: 10.1007/s00429-024-02819-y (PMC11374833; doi:10.1007/s00429-024-02819-y)
Supplement: Supplementary file 1 — Supplementary file1 (PDF 9701 KB) [file 429_2024_2819_MOESM1_ESM.pdf]

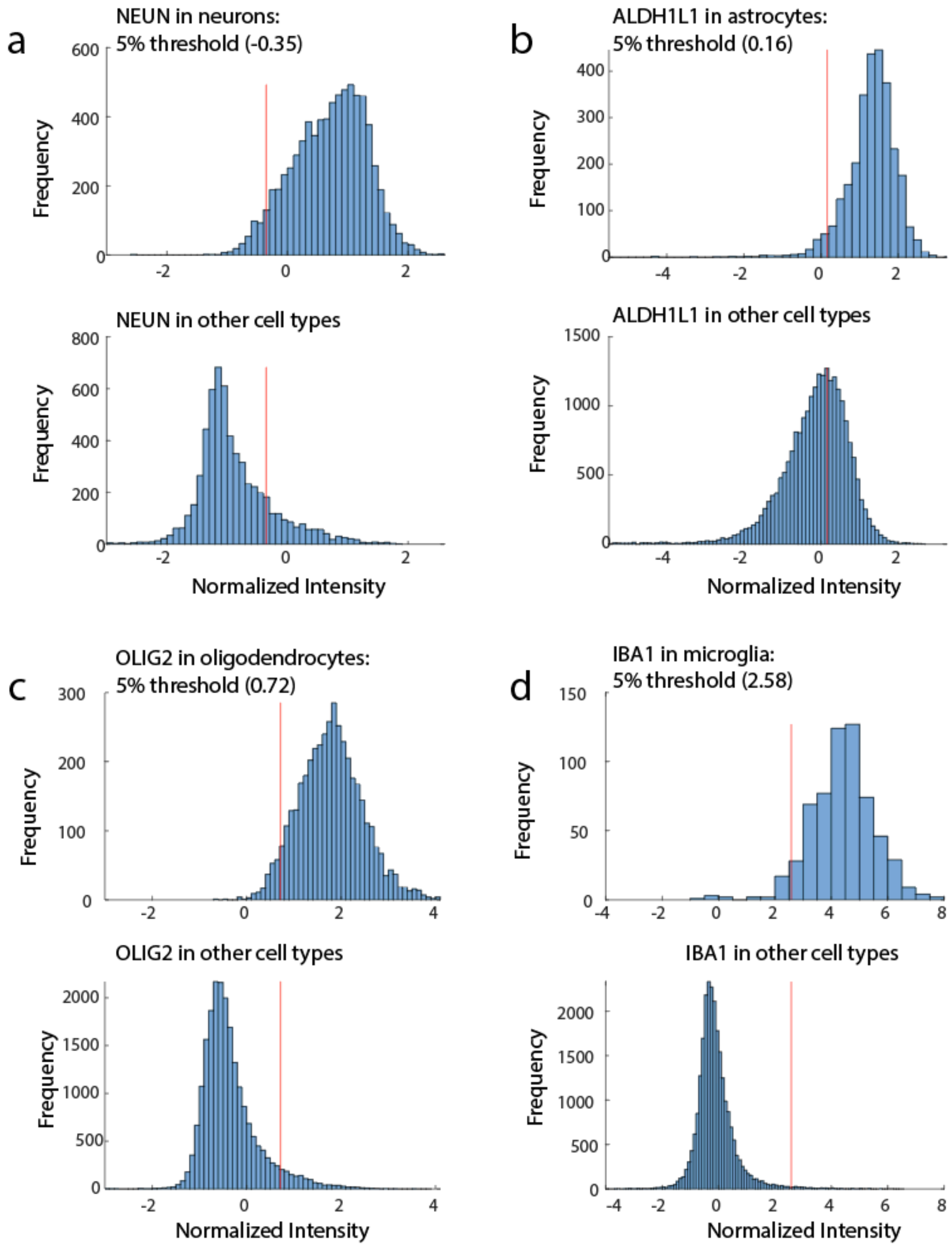

Supplementary Figure 1. Thresholding of markers that are preferentially identified in specific cell types. Histograms of the normalized grayscale intensity of markers in the reference cell type, and in all other cell types. Each of the markers shown were assumed present in 95% of the reference cell types. Red lines indicate the 5th percentile of normalized intensity in each reference cell type; any cell with normalized intensity above this threshold was labeled as positive for the corresponding marker. Distribution of **a)** NEUN within neurons (top) and other cell types; **b)** ALDH1L1 within astrocytes (top) and other cell types; **c)** OLIG2 within oligodendrocytes (top) and other cell types; **d)** IBA1 within microglia (top) and other cell types.

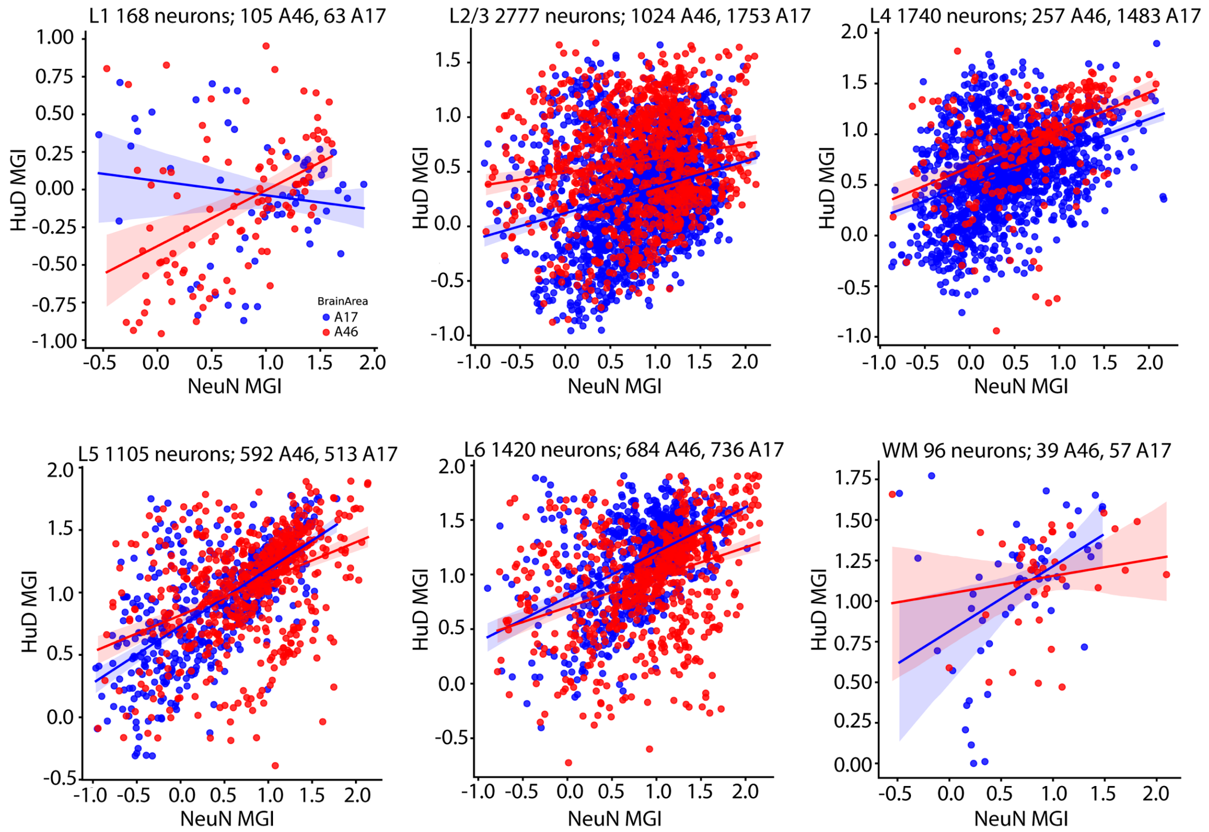

Supplementary Figure 2. HuD and NeuN mean grayscale intensity (MGI) relationships.
2-D scatter plots showing the relationships between normalized HUD intensity and normalized NEUN intensity in the total population of neurons from all subjects in L1, L2/3, L4 (top) and L5, L6, WM in A46 (red) and A17 (blue). Regression lines with translucent bars showing confidence interval are overlayed on each plot. L1: A46- R^2= 0.212, p<0.001; A17- R^2=0.020 , p=0.275; L2/3: A46- R^2=0.024, p<0.001; A17- R^2=0.067, p<0.001; L4: A46- R^2=0.213, p<0.001; A17- R^2=0.143, p<0.001; L5: A46- R^2=0.169, p<0.001; A17- R^2=0.423, p<0.001; L6: A46- R^2=0.119, p<0.001; A17- R^2=0.233, p<0.001; WM: A46- R^2=0.027, p=0.314; A17- R^2=0.191 , p<0.001

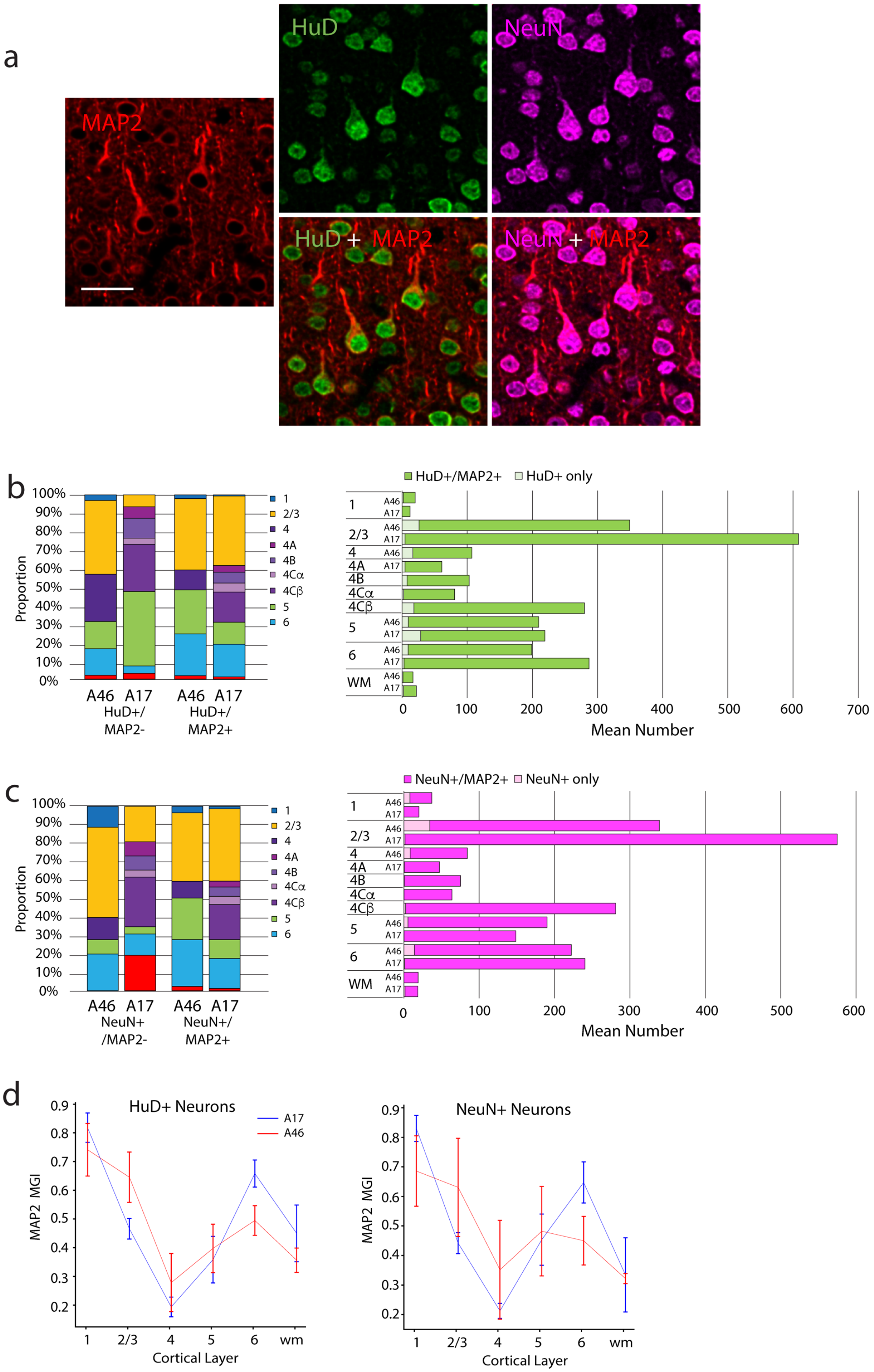
a
MAP2
HuD
NeuN
HuD + MAP2
NeuN + MAP2
b
Proportion
100%
90%
80%
70%
60%
50%
40%
30%
20%
10%
0%
1
2/3
4
4A
4B
4Cα
4Cβ
5
6
A46 A17
HuD+/
MAP2-
A46 A17
HuD+/
MAP2+
HuD+/MAP2+
HuD+ only
1
2/3
4
4A
4B
4Cα
4Cβ
5
6
WM
A46
A17
0 100 200 300 400 500 600 700
Mean Number
c
Proportion
A46 A17
NeuN+
/MAP2-
A46 A17
NeuN+/
MAP2+
NeuN+/MAP2+
NeuN+ only
0 100 200 300 400 500 600
Mean Number
d
HuD+ Neurons
NeuN+ Neurons
A17
A46
MAP2 MGI
0.9
0.8
0.7
0.6
0.5
0.4
0.3
0.2
1 2/3 4 5 6 wm
Cortical Layer

Supplementary Figure 3. Proportions and marker intensity of MAP2+/HuD+ and MAP2+/NEUN+ neurons in A46 vs. A17. **a)** Photomicrographs showing MAP2+ (red), HuD+ (green) and NeuN+ (magenta) neurons in A17 of a 19.2-year-old female monkey. Scale bar: 25μm **b)** left- graphs showing the laminar distribution of HuD+/MAP2- and HuD+/MAP2+ neurons across laminae in A46 and A17. Right- graphs showing numbers of HuD+/MAP2- and HuD+/MAP2+ neurons across laminae in A46 and A17. **c)** left- graphs showing the laminar distribution of NeuN+/MAP2- and NeuN+/MAP2+ neurons across laminae in A46 and A17. Right- graphs showing numbers of NeuN+/MAP2- and NeuN+/MAP2+ neurons across laminae in A46 and A17. **d)** Mean grayscale intensities (MGIs) of MAP2 in HuD+ (left) and in NeuN+ (right) neurons across cortical layers.

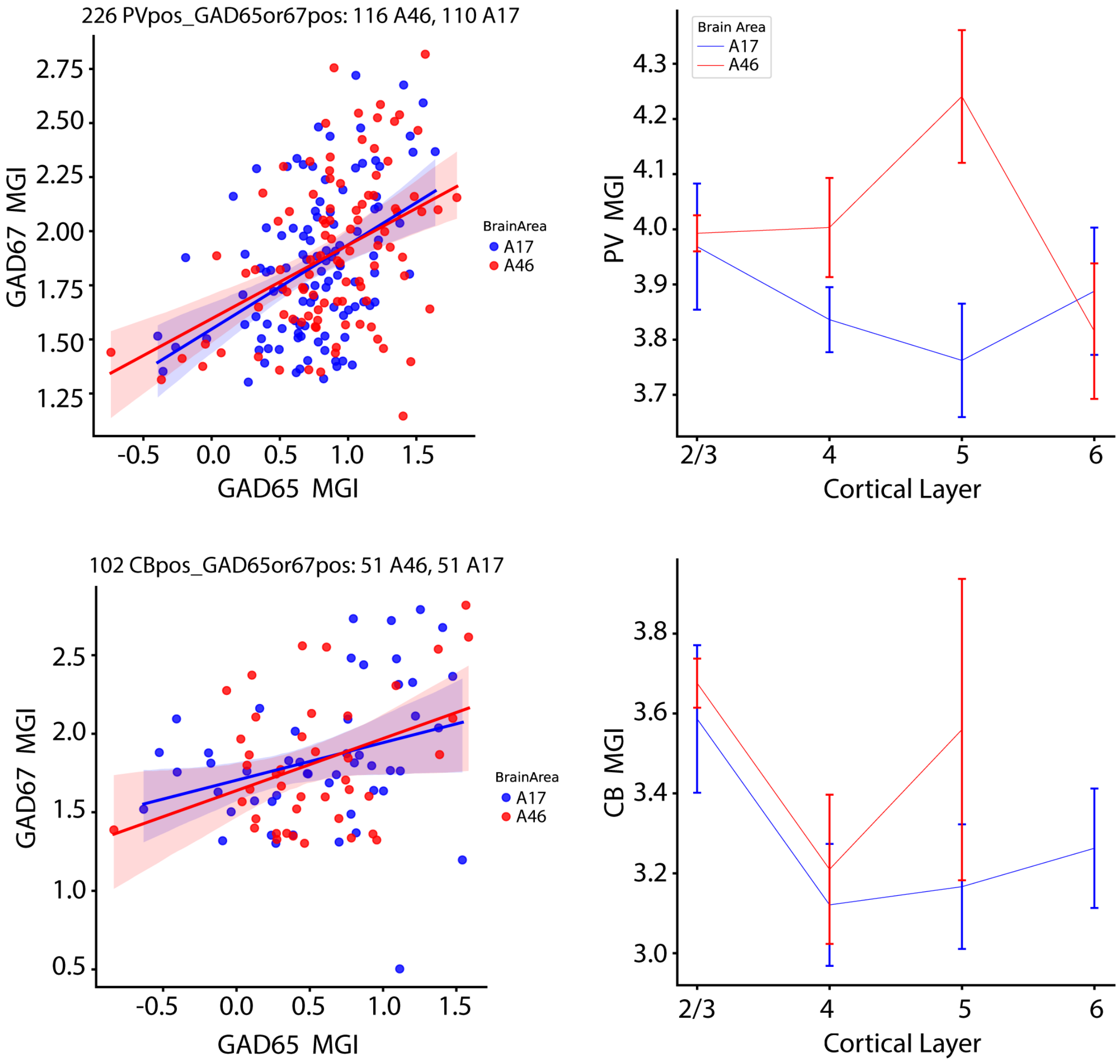

Supplementary Figure 4. Mean grayscale intensities (MGIs) of GAD67 and GAD65 in PV+ and CB+ GABAergic neurons. *Left*- 2-D scatter plots showing the relationships between normalized GAD67 intensity (CB: A46- R^2= 0.152, p=0.01; A17- R^2=0.235, p<0.001; PV: A46- R^2=0.209, p<0.001; A17- R^2=0.194, p<0.001) and *right*- normalized GAD65 intensity in the total population of PV+ (*top*) and CB+ (*bottom*) interneurons from all subjects.

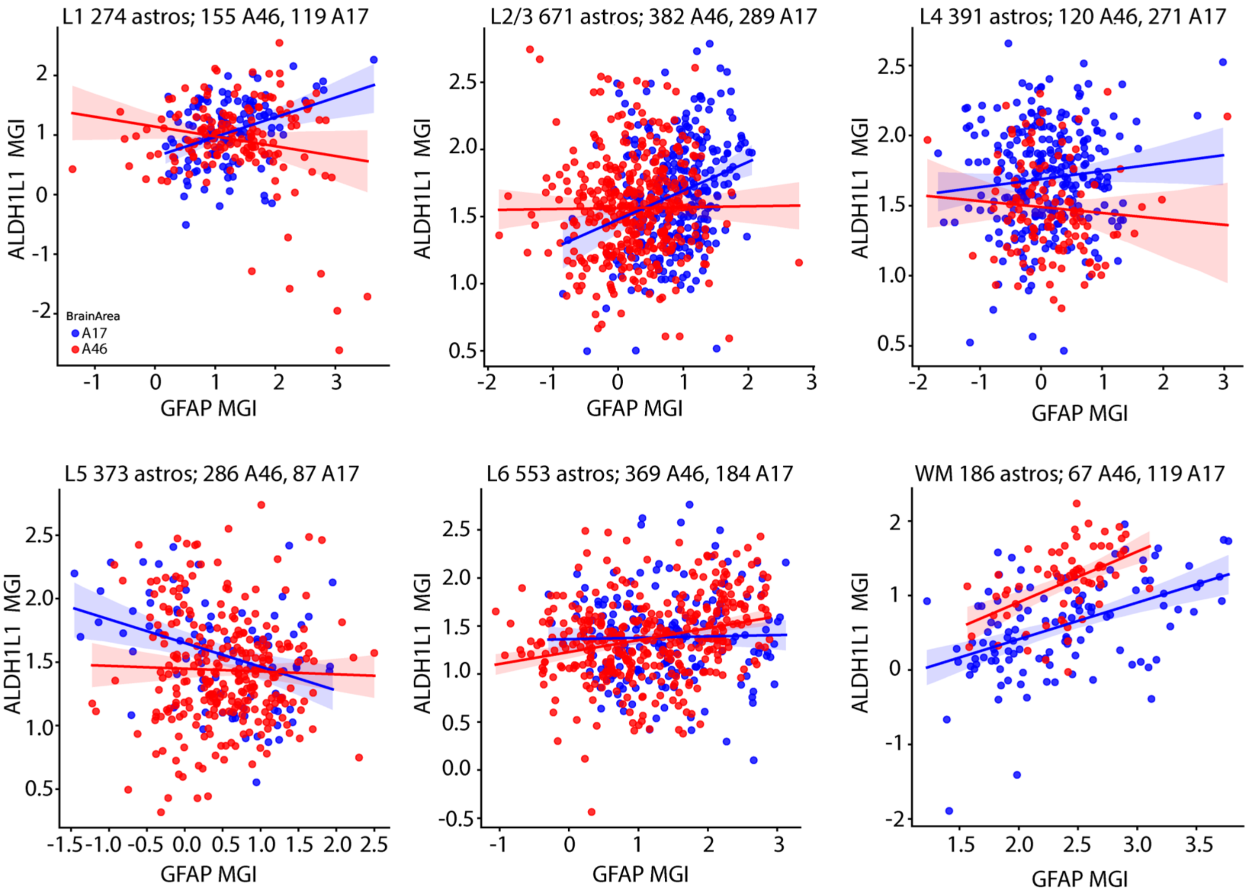

Supplementary Figure 5. ALDH1L1 and GFAP mean grayscale intensity (MGI) relationships. 2-D scatter plots showing the relationships between normalized ALDH1L1 intensity and normalized GFAP intensity in the total population of astrocytes from all subjects in L1, L2/3, L4 (top) and L5, L6, WM in 46 and A17. L1: A46- R^2=0.024287, p=0.053; A17- R^2=0.131388, p<0.001; L2/3: A46- R^2=0.001726, p=0.418; A17- R^2=0.075811, p<0.001; L4: A46- R^2=0.006383, p=0.386; A17- R^2=0.010545, p=0.092; L5: A46- R^2=0.000156, p=0.833; A17- R^2=0.129183, p<0.001; L6: A46- R^2=0.069965, p<0.001; A17- R^2=0.000583, p=0.746; WM: A46- R^2=0.247896, p<0.001; A17- R^2=0.222563, p<0.001

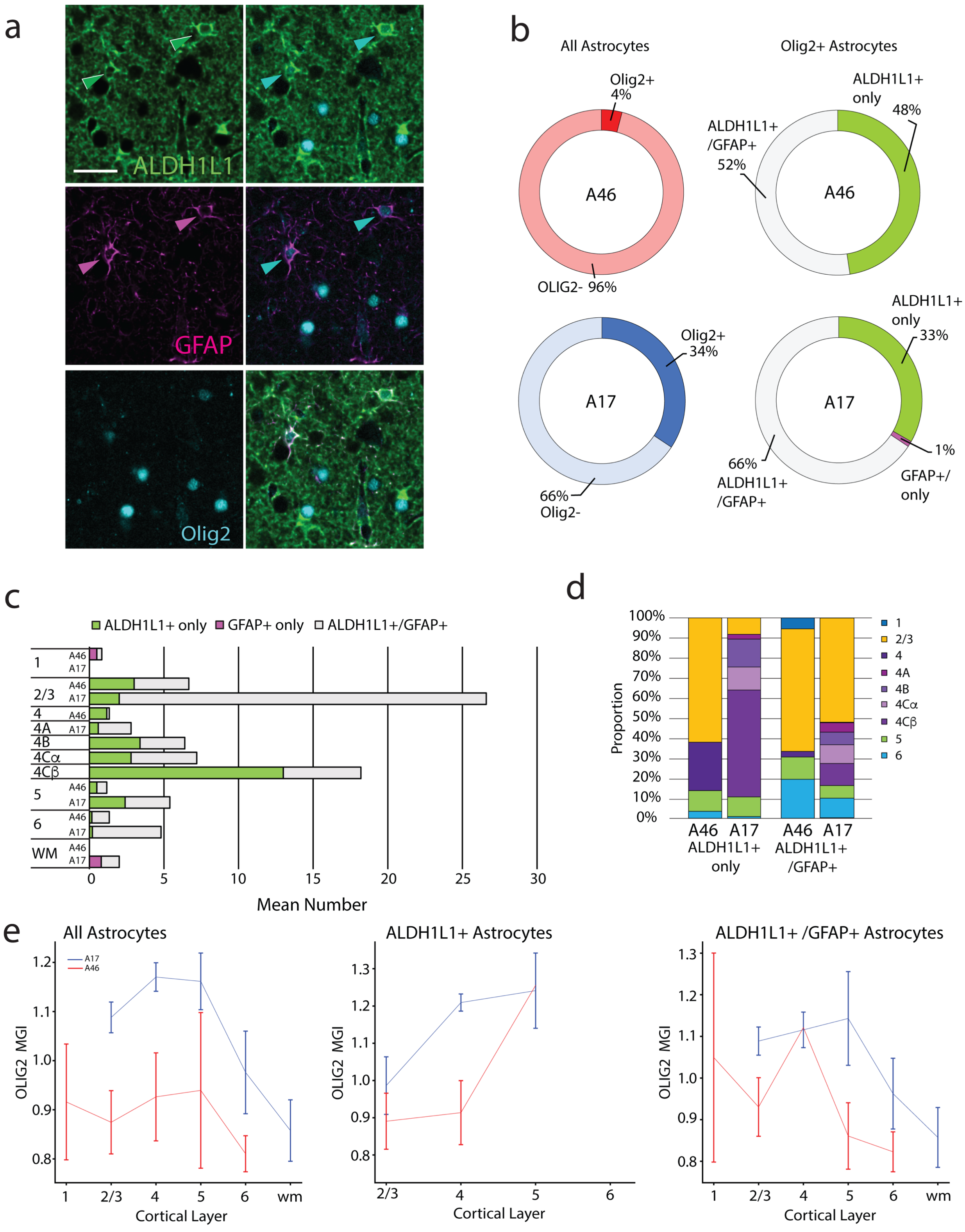
a
ALDH1L1
GFAP
Olig2
b
All Astrocytes
Olig2+ Astrocytes
Olig2+ 4%
A46
OLIG2- 96%
ALDH1L1+ only 48%
ALDH1L1+ /GFAP+ 52%
Olig2+ 34%
A17
66% Olig2-
ALDH1L1+ only 33%
1%
GFAP+/ only
66% ALDH1L1+ /GFAP+
c
ALDH1L1+ only
GFAP+ only
ALDH1L1+/GFAP+
1
2/3
4
4A
4B
4Cα
4Cβ
5
6
WM
Mean Number
d
Proportion
A46 A17 ALDH1L1+ only
A46 A17 ALDH1L1+ /GFAP+
1
2/3
4
4A
4B
4Cα
4Cβ
5
6
e
All Astrocytes
ALDH1L1+ Astrocytes
ALDH1L1+ /GFAP+ Astrocytes
A17
A46
OLIG2 MGI
Cortical Layer
wm

Supplementary Figure 6. Distribution and marker intensities in OLIG2+ astrocytes.
a) Photomicrographs showing Olig2+ in ALDH1L1+, in GFAP+ and in ALDH1L1+/GFAP+ astrocytes. Scale bar: 25μm. **b)** Ring graphs showing the relative overall proportions of Olig2+ astrocytes (total population of astrocytes) in the two brain areas (*left)* and the relative proportion of Olig2+/ALDH1L1+/GFAP+ and OLIG2+/ALDH1L1+ astrocytes in the two areas (*right*) and Olig2+/GFAP+ astrocytes in A17 (*right, bottom*). **c)** Graph showing the numbers of Olig2+/ALDH1L1+, Olig2+/GFAP+, and Olig2+/ALDH1L1+/GFAP+ astrocytes in each cortical layer. **d)** Bar graphs showing the proportions of Olig2+/ALDH1L1+/GFAP+ and OLIG2+/ALDH1L1+ astrocytes across the 6 cortical laminae. **e)** Olig2 mean grayscale intensities (MGIs) for all Olig2+ (*left*), Olig2+/ALDH1L1+ (*middle*), and Olig2+/ALDH1L1+/GFAP+ (*right*) across cortical layers.

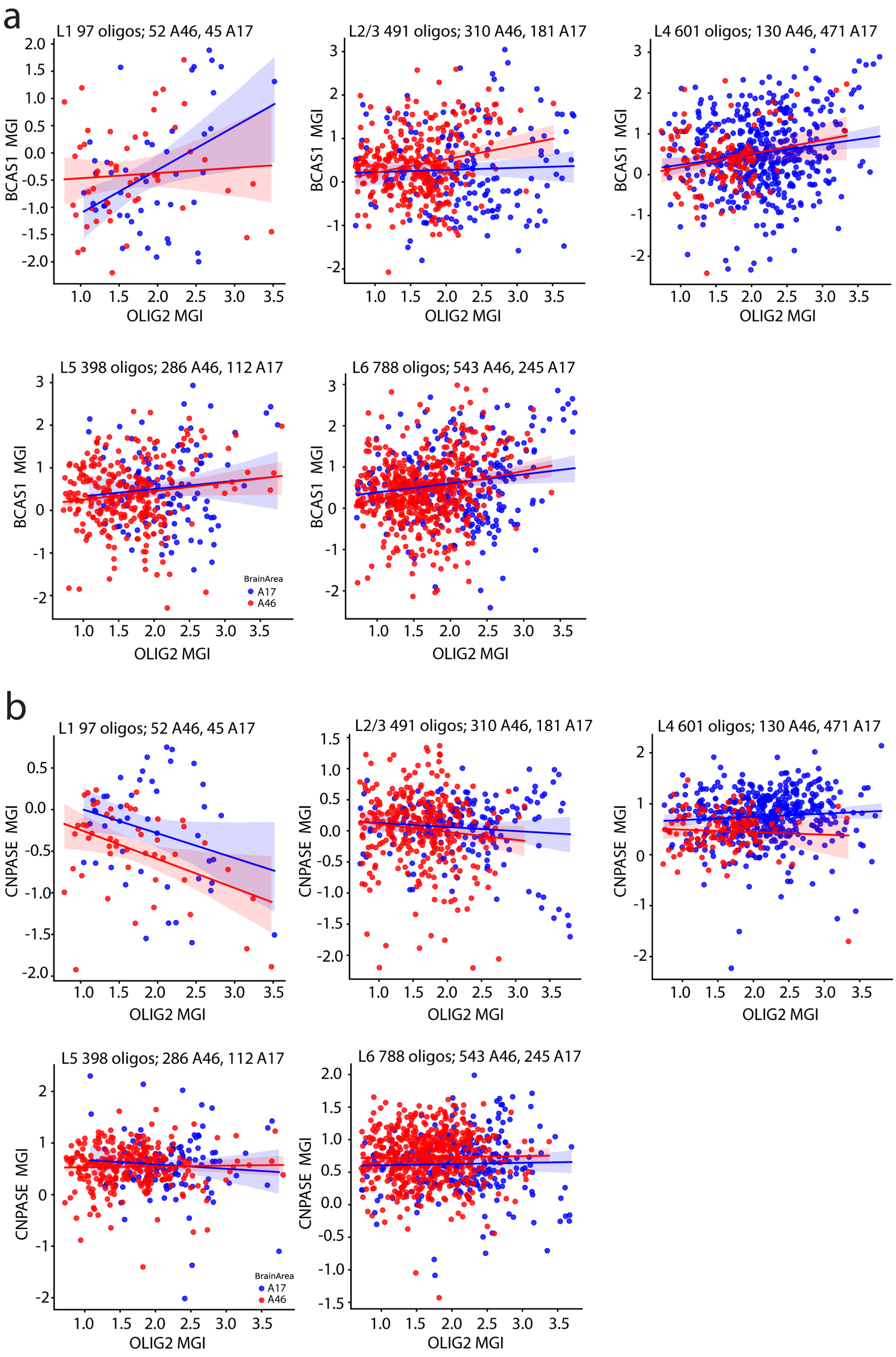
a
L1 97 oligos; 52 A46, 45 A17
L2/3 491 oligos; 310 A46, 181 A17
L4 601 oligos; 130 A46, 471 A17
L5 398 oligos; 286 A46, 112 A17
L6 788 oligos; 543 A46, 245 A17
BCAS1 MGI
OLIG2 MGI
BrainArea
A17
A46
b
L1 97 oligos; 52 A46, 45 A17
L2/3 491 oligos; 310 A46, 181 A17
L4 601 oligos; 130 A46, 471 A17
L5 398 oligos; 286 A46, 112 A17
L6 788 oligos; 543 A46, 245 A17
CNPASE MGI
OLIG2 MGI
BrainArea
A17
A46

Supplementary Figure 7. Oligodendrocyte marker laminar intensities. **a)** 2-D scatter plots showing the relationships between mean grayscale intensities (MGIs) of BCAS1 and Olig2 in A46 and A17 for all oligodendrocytes across laminae 1-6 in A17 and A46 from all subjects. **b)** 2-D scatter plots showing the relationships between BCAS1 and Olig2 MGIs in A46 and A17 for all oligodendrocytes across laminae 1-6 in A17 and A46 from all subjects. BCAS1: L1: A46- R^2=0.004945, p=0.624; A17- R^2=0.17535, p=0.004; L2/3: A46- R^2=0.044466, p<0.001; A17- R^2=0.001466, p=0.615; L4: A46- R^2=0.052894, p=0.008; A17- R^2=0.026121, p<0.001; L5: A46- R^2=0.020085, p=0.017; A17- R^2=0.009382, p=0.312; L6: A46- R^2=0.043661, p<0.001; A17- R^2=0.022511, p=0.019. CNPASE: L1: A46- R^2=0.181107, p=0.002; A17- R2=0.069892, p=0.083; L2/3: A46- R^2=0.011074, p=0.065; A17- R^2=0.008872, p=0.214; L4: A46- R^2=0.003755, p=0.489; A17- R^2=0.005627, p=0.105; L5: A46- R^2=0.000474, p=0.714; A17- R^2=0.005974, p=0.418; L6: A46- R^2=0.00026, p=0.708; A17- R^2=0.000642, p=0.693
