# Supplementary material for: Proteomic features of gray matter layers and superficial white matter of the rhesus monkey neocortex: comparison of prefrontal area 46 and occipital area 17
Source: Brain Struct Funct. 2024 Jun 28;229(7):1495–525. doi: 10.1007/s00429-024-02819-y (PMC11374833; doi:10.1007/s00429-024-02819-y)
Supplement: Supplementary file 2 — Supplementary file2 (PDF 61 KB) [file 429_2024_2819_MOESM2_ESM.pdf]

Supplementary Table 1. Antibodies and supplemental reagents used in this study.

Primary Unconjugated Antibodies

Target	Primary Ab Vendor & Catalog #	Clone	Isotype	Primary Ab Working Concentration (ug/mL)	Secondary Ab Vendor & Catalog #	Dye type	Secondary Ab Working Concentration (ug/mL)
3-nitrotyrosine	Abcam, ab61392	39B6	Mouse IgG2ak	10	Jackson Immuno, 715-606-150	Cy5	5
GAD67	R&D Systems, AF2086	Polyclonal	Goat IgG	2.5	Jackson Immuno, 705-225-147	Cy2	5
TSPO	Abcam, ab213654	Polyclonal	Rabbit IgG	5	Jackson Immuno, 711-166-152	Cy3	5

In-House Dye Labeled Antibodies

Target	Ab Vendor & Catalog #	Clone	Isotype	Dye type	D:P ratio	Working Concentration (ug/mL)
Abeta	Biolegend, 803001	6E10	Mouse IgG1	Cy3	3.4	5
ALDH1L1	Millipore, MABN495	N103-39	Mouse IgG1	Cy3	5.8	5
pSer202/pThr205 Tau	Thermo, MN1020	AT8	Mouse IgG2	Cy5	1.5	5
Caspase3, cleaved	Cell Signaling, 9664BF	5A1E	Rabbit IgG	Cy5	3.2	5
Collagen IV	Millipore, MAB3326	IV-4H12	Mouse IgG1k	Cy3	3.6	5
CNPase	Cell Signaling, 5664	D83E10	Rabbit IgG	Cy5	5.0	5
GAD65	Cell Signaling, 5843	D5G2	Rabbit IgG	Cy2	4.2	5
Iba1	Wako chemical, 019-19741	Poly	Rabbit IgG	Cy5	1.3	5
MAP2	Sigma, M4403	HM-2	Mouse IgG1	Cy5	2.1	5
MBP	Abcam, ab7349	12	Mouse IgG2a	Cy2	4.5	5
NeuN	Millipore, MAB377	A60	Mouse IgG1	AF647	2.56	5
Parvalbumin	Swant, PV 235	PV 235	Mouse IgG1	Cy3	5.2	5
TDP-43	Proteintech, 10782-2-AP	Poly	Rabbit IgG	Cy3	3.4	10

Commercial Purchased Dye Labeled Antibodies

Target	Ab Vendor & Catalog #	Clone	Isotype	Dye type	Working Concentration (ug/mL)
GFAP	Sigma, C9205	G-A-5	Mouse IgG2	Cy3	1
HUD	Santa Cruz, sc-28299	E-1	Mouse IgG2a	AF647	10
Olig2	Abcam, ab225100	EPR2673	Rabbit IgG	AF647	10
pNF	BioLegend, SMI-31P	SMI-31	Mouse IgG1	Cy3	5
S6	Santa Cruz, sc-74459	C-8	Mouse IgG2b	Cy5	5
SMA	Sigma, C6198	1A4	Mouse IgG2a	Cy3	2.5
Vimentin	Cell Signaling, 9854	D21H3	Rabbit IgG	AF488	1

Zenon Labeled Antibodies

Target	Primary Ab Vendor & Catalog #	Clone	Zenon Kit Vendor & Catalog #	D:P ratio	Dye type	Working Concentration (ug/mL)
BCAS1	Abcam, ab106661	Poly	ThermoFisher, Z25308	3	AF647	10
Calbindin	Swant, CB38	CB38	ThermoFisher, Z25308	3	AF647	10
Collagen IV	Novus, NBP1-26549	Poly	ThermoFisher, Z25608	3	AF647	10
Kv3.1	Millipore, AB5188	Poly	ThermoFisher, Z25308	3	AF647	5
npNF	BioLegend, SMI-32P	SMI-32	ThermoFisher, Z25005	3	AF555	10
pan-pNF	Biolegend, 837904	SMI-312	ThermoFisher, Z25002	3	AF488	10

Supplemental reagents

Item	Vendor	Catalog #
Phosphate Buffered Saline	Lonza	BE17-517Q
Vector Laboratories Antigen Unmasking Solution, Citric Acid Based	ThermoFisher	H-3300-250
Bovine Serum Albumin	Sigma	A2153
Normal Donkey Serum	Jackson	017-000-121
Normal Rabbit Serum	Jackson Immuno	011-000-120
Normal Mouse Serum	Jackson Immuno	015-000-120
Normal Goat Serum	Jackson Immuno	005-000-121
Normal Guinea Pig Serum	Jackson Immuno	006-000-120
DAPI (4',6-Diamidino-2-Phenylindole, Dilactate)	ThermoFisher	D3571
Lecia Bond™ Research Detection System	Leica Biosystems	DS9455
Leica Bond™ Wash Solution 10x Concentrate	Leica Biosystems	AR9590
Zenon™ Alexa Fluor™ Mouse IgG1 Labeling Kit, AF488	ThermoFisher	Z25002
Zenon™ Alexa Fluor™ Mouse IgG1 Labeling Kit, AF555	ThermoFisher	Z25005
Zenon™ Alexa Fluor™ Mouse IgG1 Labeling Kit, AF647	ThermoFisher	Z25008
Zenon™ Alexa Fluor™ Rabbit IgG Labeling Kit, AF555	ThermoFisher	Z25305
Zenon™ Alexa Fluor™ Rabbit IgG Labeling Kit, AF647	ThermoFisher	Z25308
Zenon™ Alexa Fluor™ Goat IgG Labeling Kit, AF647	ThermoFisher	Z25608
Anti-Guinea Pig IgG (H+L), highly cross-adsorbed, CF™555 antibody	Sigma	SAB4600297
Cy™2 AffiniPure Donkey Anti-Goat IgG (H+L)	Jackson ImmunoResearch	705-225-147
Cy™3 AffiniPure $F(ab')_2$ Fragment Donkey Anti-Rabbit IgG (H+L)	Jackson ImmunoResearch	711-166-152
Alexa Fluor® 647 AffiniPure $F(ab')_2$ Fragment Donkey Anti-Mouse IgG (H+L)	Jackson ImmunoResearch	715-606-150
