# Supplementary material for: Proteomic features of gray matter layers and superficial white matter of the rhesus monkey neocortex: comparison of prefrontal area 46 and occipital area 17
Source: Brain Struct Funct. 2024 Jun 28;229(7):1495–525. doi: 10.1007/s00429-024-02819-y (PMC11374833; doi:10.1007/s00429-024-02819-y)
Supplement: Supplementary file 3 — Supplementary file3 (PDF 35 KB) [file 429_2024_2819_MOESM3_ESM.pdf]

LAYER	A46	A17
**1**	0-7%	0-6%
**2/3**	7-40%	6-36%
**4**	40-50%	36-68%*
**5**	50-71%	68-79%
**6**	71-100%	79-100%

Supplementary Table 2. Laminar proportions as percent distance from pia. *Sublayers 4A, 4B, 4Cα, and 4Cβ were 10, 20, 20 and 50%, respectively.
