# Supplementary material for: Proteomic features of gray matter layers and superficial white matter of the rhesus monkey neocortex: comparison of prefrontal area 46 and occipital area 17
Source: Brain Struct Funct. 2024 Jun 28;229(7):1495–525. doi: 10.1007/s00429-024-02819-y (PMC11374833; doi:10.1007/s00429-024-02819-y)
Supplement: Supplementary file 4 — Supplementary file4 (PDF 24 KB) [file 429_2024_2819_MOESM4_ESM.pdf]

Supplementary Table 3. Threshold values for definition of marker positivity within cells.

Marker	Reference Cell Type	Cellular region	Threshold Percentile
ALDH1L1	astrocyte	both	5%
GFAP	astrocyte	both	20%
Iba1	microglia	nucleus	5%
HUD	neuron	both	5%
MAP2	neuron	cytoplasm	5%
NeuN	neuron	both	5%
GAD65	neuron	both	90%
GAD67	neuron	both	90%
Kv3.1	neuron	both	90%
CB	neuron	both	98%
PV	neuron	both	98%
Olig2	oligo	nucleus	5%
CNPase	oligo	cytoplasm	50%
BCAS1	oligo	both	95%
